# Genome-driven evolutionary game theory helps understand the rise of metabolic interdependencies in microbial communities

**DOI:** 10.1038/s41467-017-01407-5

**Published:** 2017-11-16

**Authors:** Ali R. Zomorrodi, Daniel Segrè

**Affiliations:** 1 Bioinformatics Program, Boston University, Boston, MA 02215 USA; 2 Department of Biology, Boston University, Boston, MA 02215 USA; 3 Biological Design Center, Boston University, Boston, MA 02215 USA; 4 Department of Biomedical Engineering, Boston University, Boston, MA 02215 USA

## Abstract

Metabolite exchanges in microbial communities give rise to ecological interactions that govern ecosystem diversity and stability. It is unclear, however, how the rise of these interactions varies across metabolites and organisms. Here we address this question by integrating genome-scale models of metabolism with evolutionary game theory. Specifically, we use microbial fitness values estimated by metabolic models to infer evolutionarily stable interactions in multi-species microbial “games”. We first validate our approach using a well-characterized yeast cheater-cooperator system. We next perform over 80,000 in silico experiments to infer how metabolic interdependencies mediated by amino acid leakage in *Escherichia coli* vary across 189 amino acid pairs. While most pairs display shared patterns of inter-species interactions, multiple deviations are caused by pleiotropy and epistasis in metabolism. Furthermore, simulated invasion experiments reveal possible paths to obligate cross-feeding. Our study provides genomically driven insight into the rise of ecological interactions, with implications for microbiome research and synthetic ecology.

## Abstract (web summary)

The rise of metabolic interdependencies among microbes is still poorly understood. Here, taking the underlying biochemical networks into consideration, Zomorrodi and Segrè integrate genome-scale metabolic models with evolutionary game theory to study the rise of cross-feeding in microbial communities.

## Introduction

Obligate dependencies among microorganisms through the exchange of essential metabolites have been hypothesized to be ubiquitous in microbial ecosystems^1,2^. Similar interactions have also been engineered in laboratory systems, mainly based on genetically induced auxotrophies^3–8^. However, the evolutionary rise and maintenance of these interactions constitutes an unresolved puzzle, since genotypes that do not contribute to the production of costly metabolites may have a selective advantage over producers. One theory, known as the Black Queen (BQ) Hypothesis^9^, suggests that in communities with BQ functions (essential functions that are costly to focal cells, or “producers”, but are unavoidably leaky and partially available to the broader community) metabolic dependencies could arise through adaptive gene loss: in such communities organisms benefit from losing their own capacity to produce a costly metabolite (thus becoming “non-producers”). This could give rise to an obligate dependency of non-producers on producers^9^, or, in the case of more than one BQ function, to obligate cross-feeding (bidirectional dependency)^10^. However, little is known about the conditions under which these dependencies would be established, as the rise of mutant genotypes due to adaptive gene loss does not necessarily guarantee a stable coexistence.

A limited number of theoretical studies have recently explored this question using ecological models^11–14^. Similarly, other studies have used evolutionary game theory (see refs. ^15–18^ for comprehensive reviews), and concepts from economics^19^ to better understand inter-species dependencies in microbial communities. While these approaches have provided valuable phenomenological insight into the general principles of metabolic interdependencies, they often do not take into account the specific details of the organisms, pathways, and molecules involved: behind the biosynthesis, leakiness, and utilization of these metabolites, is a complex network of biochemical reactions, which may significantly vary across different environmental conditions, metabolites, and organisms. A powerful avenue to address this gap is the use of systems biology methods, such as genome-scale network models of metabolism^20^. These models take into account the full metabolic circuitry of a cell and provide quantitative predictions of its growth capacity and metabolic fluxes. Recent work has started applying these approaches to model microbial communities^21–30^ (also see ref. ^31^ for a recent review) and to study the evolution of adaptive diversification in long-term evolutionary experiments^32^. However, a systematic analysis of the possible equilibrium states of interacting species as a function of the leakiness of various metabolites, and of their underlying metabolic circuits is still lacking.

Here we propose a hybrid modeling approach that combines the theoretical insight of evolutionary game theory with the organism-specific-detailed analysis of cell-wide metabolic networks. We demonstrate how this strategy allows one to map the landscape of possible inter-species interactions, for which genome-scale metabolic models provide unique mechanistic insights. In addition to providing a genomic- and biochemistry-grounded basis for the quantitative assessment of the BQ Hypothesis, our approach can generate testable organism- and metabolite-specific predictions of inter-species interactions equilibria.

## Results

### Integrating metabolic networks and evolutionary game theory

**Fig. 1:**
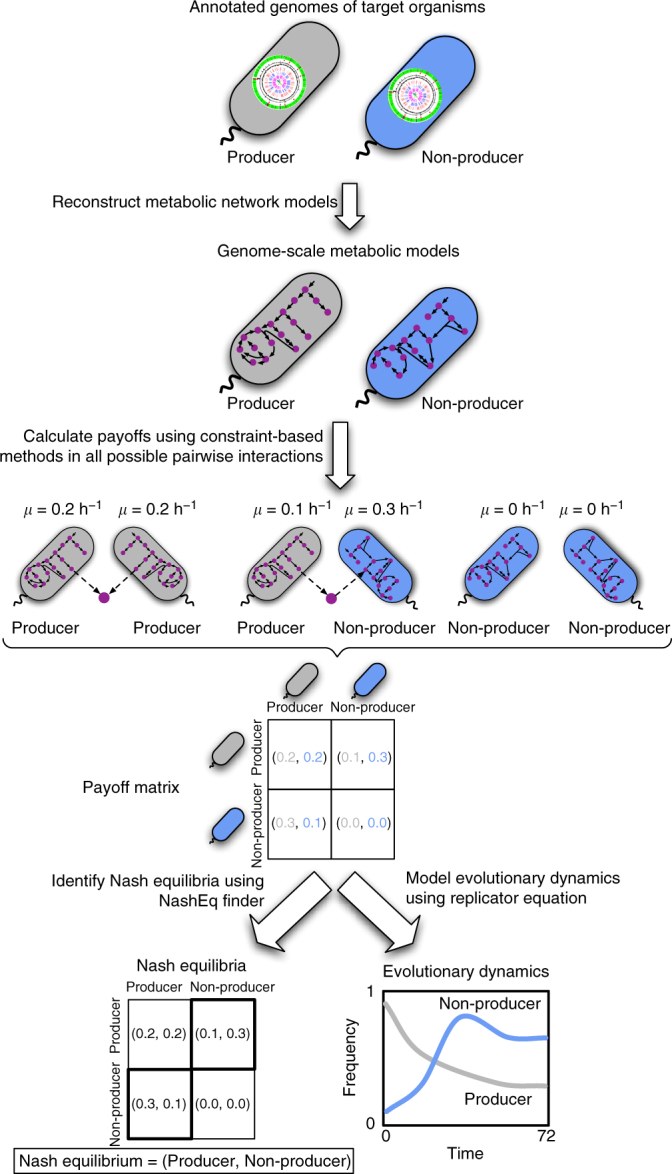
General scheme for the proposed genome-driven evolutionary game theory approach. Annotated genomes of community members are used to construct genome-scale metabolic models. For each possible pair of genotypes in the community, constraint-based analysis tools for metabolic models, such as flux balance analysis^51^, are used to estimate the fitness (or “payoff”) of each genotype as they engage in a specific metabolite-mediated interaction. These payoffs form the payoff matrix of the game. Based on this payoff matrix, we identify all pure strategy Nash equilibria of the game, using an automated pipeline (NashEq Finder, see “Methods”). The payoff matrix also allows one to model evolutionary dynamics (i.e., how genotype frequencies change over time)^33^ and to determine which of the identified Nash equilibria are evolutionarily stable (see “Methods”). Supplementary Figs. 11 and 12 provide a more specific representation of this scheme for the presented case studies in this paper

Our genomically-driven game theory approach enables a fast way of computing physiologically relevant estimates of the fitness (or “payoff”) of microbes involved in metabolic interactions, and of inferring the evolutionarily stability of such interactions under diverse environmental or strategic conditions. Different microbes, identified here with their genotypes, are assumed to potentially leak specific metabolites that can be utilized by other community members. For each possible pair of genotypes in the community, we used constraint-based analysis of genome-scale metabolic models to estimate their fitness (payoff) as they engage in a specific metabolite-mediated interaction. The payoff of a genotype is set to its predicted growth rate, implicitly taking into account how this rate will be affected by the level of leakage and by the biosynthesis and possible transport cost of the specific metabolite(s) leaked (see “Methods” and Fig. 1). From these estimated payoffs (forming the “payoff matrix” of the game), we can infer which pairs of interacting genotypes can equilibrate in the community and drive its fate. This is achieved by using the game theory concept of “Nash equilibrium”, defined as a state where no player can increase its payoff by a unilateral change of strategy. To automate the identification of Nash equilibria for a game involving two or more players, we developed an optimization-based algorithm called NashEq Finder (see “Methods”). Note that in this algorithm, and throughout this work, we focus only on pure strategy Nash equilibria^33^. The payoff matrix also allows one to model evolutionary dynamics (i.e., how genotype frequencies change over time)^33^ and to determine which of the identified Nash equilibria are evolutionarily stable (see “Methods” and Fig. 1). It is worth noting that, while constraint-based analyses of metabolic networks often seek the prediction of optimal states (e.g., by maximizing growth), their integration with evolutionary game theory enables the inference of non-optimal equilibrium states of a microbial community.

### Metabolic dependencies in invertase-producing *S*. *cerevisiae*

**Fig. 2:**
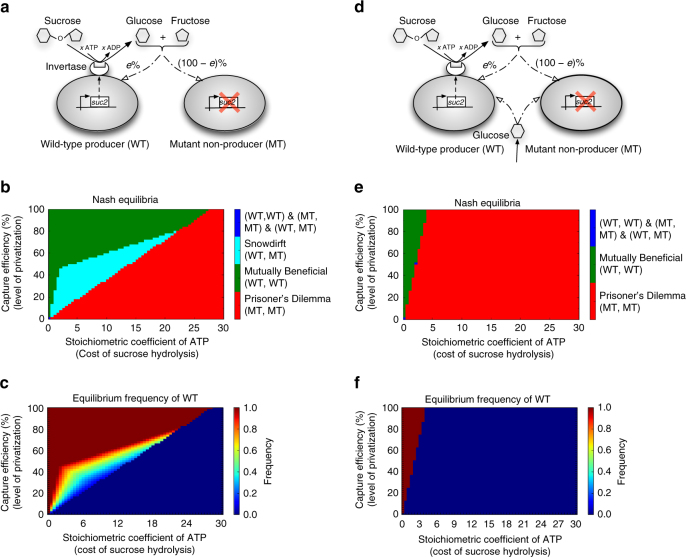
Metabolic dependencies in populations of *S. cerevisiae* growing on sucrose. **a** Metabolic interactions between producer (wild-type, WT) and non-producer (mutant, MT) genotypes of *S*. *cerevisiae* growing on sucrose^34^. Here, *e* represents the percentage of glucose/fructose that diffuses away and serves as a public good. **b** Nash equilibria and **c** the equilibrium frequency of WT for the community shown in **a** as a function of the capture efficiency of the glucose/fructose and the invertase production cost (that latter was implicitly modeled by changing the stoichiometric coefficient of ATP in the sucrose hydrolysis reaction, indicated by *x*). An alternative in silico formulation of the energetic cost of invertase production that reproduces exactly the setup used in the experiment by Gore et al.^34^ (based on histidine auxotrophy) proved to be qualitatively equivalent to the analysis presented here (see Supplementary Note 2 for details). The equilibrium frequency of WT in **c** was obtained from in silico invasion experiments (see “Methods”) for two cases of a small fraction of MT invading a resident population of WT and vice versa. This analysis demonstrated that the equilibrium frequency of WT is the same in both cases (results are shown here for only one case). **d** Metabolic interactions between WT and MT when additional glucose is provided in the growth medium (see Supplementary Methods for details of implementation). **e** Nash equilibria and **f** the equilibrium frequency of WT in the presence of glucose in the growth medium. The entire Snowdrift game region and part of the Mutually Beneficial region in **b** are replaced by the Prisoner’s Dilemma game, in **e**, which is consistent with previous reports^34^ and serves as an additional verification of our modeling approach. This is because in the presence of an external supply of glucose, MT is less dependent on WT, leading to an increase in the average fitness of MT

As a proof of concept of our approach, we sought to reproduce the experimentally observed equilibria in the well-characterized yeast sucrose hydrolysis system^34^: when growing on sucrose, *S*. *cerevisiae* produces the surface enzyme invertase (encoded by the *suc2* gene), hydrolyzing sucrose into glucose and fructose, part of which leak out and serve as a public good. Since invertase production is energetically costly, a mutant strain, which has lost its *suc2* gene, may emerge (Fig. 2a). Given that this non-producer mutant strain does not incur the production of invertase and just reaps the benefit of public goods, it can reproduce faster and eventually dominate the co-culture thereby leading to the collapse of the community (a scenario called Prisoner’s Dilemma in game theory). It was shown, however, that, in addition to the Prisoner’s Dilemma, alternative outcomes are possible in certain ranges of tunable parameters, i.e., the cost of invertase production and the sugars capture efficiency (the percentage of glucose and fructose not lost in the form of public goods)^34^. These alternative outcomes include the Mutually Beneficial game (where producers dominate), and the Snowdrift game (where producers and non-producers coexist)^34^.

We constructed the in silico producer and non-producer strains using the *i*AZ900 yeast metabolic model^35^, and inferred the Nash equilibria of the system as a function of the glucose/fructose capture efficiency and the invertase production cost (see “Methods” and Supplementary Methods for details of flux balance analysis formulations to estimate the payoffs). Variations in the invertase production cost were implicitly modeled by changing the stoichiometric coefficient of ATP in the sucrose hydrolysis reaction in the metabolic model. We thus explored systematically how the Nash equilibria of the system vary over a grid of possible values of the capture efficiency and the invertase production cost. This analysis showed that all three types of Nash equilibria observed experimentally^34^ could be reproduced (Fig. 2b): Prisoner’s Dilemma emerges at high costs of sucrose hydrolysis or low glucose/fructose capture efficiency, a Mutually Beneficial game occurs at low costs of sucrose hydrolysis and high glucose/fructose capture efficiencies, and a Snowdrift game for values in between. As shown in Fig. 2c, in silico invasion experiments also recapitulate the experimental observation that the equilibrium frequency of the producers and non-producers do not depend on their initial frequencies. As an additional validation of our approach, we reproduced, in silico, the experimental observation^34^ that addition of extra glucose to the growth medium leads to a decrease in the equilibrium fraction of producers and their eventual extinction (see Fig. 2d–f for details). Thus, based entirely on the detailed knowledge of an organism’s metabolic network, which allowed us to estimate payoffs in inter-microbial games, our pipeline can reproduce known Nash equilibria previously observed experimentally and identified through phenomenological models that depend on ad hoc nonlinearity assumptions^34^.

### Single amino acid dependencies in *E*. *coli*

**Fig. 3:**
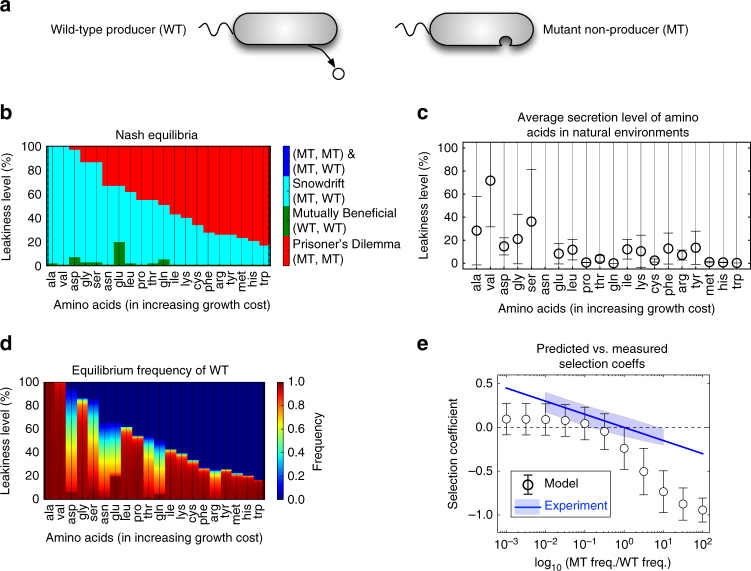
Metabolic dependencies in populations of *E. coli* secreting one amino acid. **a** Possible genotypes in populations of *E*. *coli* leaking an amino acid include a prototrophic wild-type strain (WT) self-synthesizing a leaky amino acid and a mutant strain (MT) lacking the gene(s) for the biosynthesis of this amino acid. **b** The identified Nash equilibria for various leakiness levels (as a percentage of an in silico determined maximum: see Supplementary Methods) across all 20 amino acids. Amino acids are shown here by using their standard three-letter code in the order of increasing in silico growth cost (see also Supplementary Fig. 1). **c** Experimentally reported leakiness levels of amino acids averaged over three different data sets^37,38^. Values in each data set were normalized to their maximum (see Supplementary Data 2 for values of data). Error bars show standard deviation over the three data sets. **d** The equilibrium frequency of WT as a function of the leakiness level and amino acid type. In silico invasion experiments for two cases of MT invading WT and WT invading MT revealed that the equilibrium frequencies are insensitive to the initial frequencies (results are shown here for only one case). **e** Predicted selection coefficients across all amino acids and leakiness levels vs. the experimentally reported ones for *E*. *coli* ^39^. Empty circles and error bars show, respectively, the average and the range (i.e., minimum and maximum) of the computed selection coefficients across all amino acids and leakiness levels. The blue line and the shaded region around it show the line fitted to experimentally measured selection coefficients and their range (from ref. ^39^, amino acid-deficient regime), respectively

Upon verifying our approach in the yeast sucrose hydrolysis system, we sought to characterize the landscape of possible ecological interactions in a different microbial system consisting of a large set of strains and exchanged metabolites. In particular, we explored the establishment of metabolic dependencies mediated by the leakiness of individual amino acids in *E*. *coli* and asked how these dependencies vary across the 20 amino acids and different leakiness levels. Here, a prototrophic wild-type (WT, producer), leaking a given amino acid could interact with a mutant strain (MT, non-producer) lacking the gene(s) for the biosynthesis of that amino acid (Fig. 3a). The *i*JO1366 genome-scale model of *E*. *coli* ^36^ was used to construct in silico producer and non-producer strains (see Supplementary Methods and Supplementary Data 1 for details). Given that we do not know what level of leakiness may be manifested for each amino acid in natural *E*. *coli* strains and communities, we explored the expected equilibria of this system for any leakiness level (within a sensible range) across different amino acids. With this analysis (as done before in the yeast case, see Supplementary Table 1 for a side-by-side comparison of the two systems), we systematically study the BQ Hypothesis over a large grid of two of its key parameters, i.e., the cost of performing a BQ function and the level of leakiness.

As shown in Fig. 3b, Prisoner’s Dilemma, Mutually Beneficial, and Snowdrift outcomes are the major possible equilibria, similar to the yeast system (Fig. 2b, where the amino acid cost is analogous to the sucrose hydrolysis cost and the leakiness level plays a role similar to the capture efficiency; see also Supplementary Table 1). However, a more complex pattern is observed here for the Mutually Beneficial region (see Supplementary Note 3), highlighting the organism- and product-specific nature of our approach. In addition, one can observe that, for each amino acid, there is a threshold for leakiness level above which non-producer mutants (MT) dominate, thereby leading to community collapse (i.e., Prisoner’s Dilemma) (Fig. 3b). Thus, we may expect the amino acids’ secretion levels by *E*. *coli* in natural microbial communities to lie below this threshold. While measurements for directly testing this prediction are not currently available, the average of multiple published amino acid secretion data sets^37,38^ (Fig. 3c) displays a consistent trend of leakiness levels decreasing with increasing amino acids biosynthesis cost.

To assess the evolutionary stability of these Nash equilibria, we performed in silico invasion experiments, where a resident population of WT is invaded by a low-frequency MT and vice versa. This analysis showed that the equilibrium frequencies of WT and MT are independent of their initial frequencies (Fig. 3d). This has been theoretically and experimentally suggested^10,39^ to stem from the negative frequency dependence of fitness. In addition to recapitulating this pattern, our analysis provides a quantitative prediction of the selection coefficients of the 20 amino acids. As shown in Fig. 3e, the predicted selection coefficients are within the range of data available from previous experimental reports for *E*. *coli* ^39^ for MT/WT frequency ratios of less than one. However, the model underestimates the selection coefficients for frequency ratios greater than one, and displays deviations from linearity at its extremes, where experimental data are not currently available. Overall, this analysis underscores that our approach can provide a realistic quantitative link between metabolic circuitry and important ecological parameters.

### A global map of interactions mediated by amino acid pairs

**Fig. 4:**
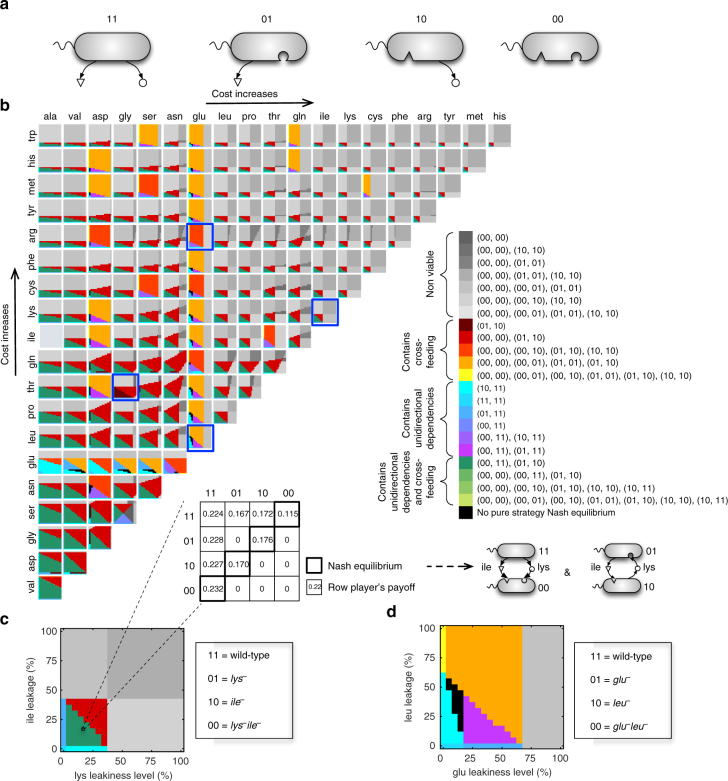
Equilibrium metabolic dependencies in populations of *E. coli* with two leaky amino acids. **a** Genotypes involved include a prototrophic strain self-synthesizing two leaky amino acids (i.e., 11), two single-mutant strains each is auxotrophic for one amino acid but synthesizing and leaking the other (i.e., 01 and 10), and a mutant strain auxotrophic for both leaky amino acids (i.e., 00). Here, “1” and “0” denote the presence or absence of biosynthesis pathways (genes) for an amino acid, respectively. **b** The identified Nash equilibria of two-player games (i.e., pairwise interactions) for all amino acid pairs across different leakiness levels, zoomed in for two selected pairs including **c** lysine and isoleucine, **d** glutamate and leucine. A sample payoff matrix of the game is shown in **c** for a leakiness level of 15% for both lysine and isoleucine. Non-viable equilibria signify associations between genotypes that are non-viable leading to community collapse. Nash equilibria of three- and four-player games for a selected number of amino acid pairs are also given in Supplementary Figs. 2–7

We further extended our analysis to map the landscape of ecological interactions in populations of *E*. *coli* strains with two leaky amino acids. Under what conditions would the increased number of exchangeable metabolites give rise to more complex inter-species interdependencies, such as reciprocal exchanges (cross-feeding)? Four different genotypes are possible in this case (Fig. 4a): a prototrophic genotype that produces and leaks two amino acids (i.e., a full producer, denoted as “11”), two partial producer mutants (denoted as “01” and “10”) each auxotrophic for one amino acid (due to loss of the corresponding biosynthesis genes) but synthesizing and leaking the other amino acid, and a no-producer mutant strain (denoted as “00”) that is auxotrophic for both amino acids (due to loss of both amino acid biosynthesis genes). Here, “1” and “0” denote the presence or absence of biosynthesis pathways (genes) for an amino acid, respectively. In a community composed of all these four genotypes, different types of interactions are possible. Here we focus on pairwise interactions, such as cross-feeding, [01, 10], and unidirectional dependency, [00, 11], though higher-order interactions among three or four genotypes (e.g., [00, 01, 10] and [00, 01, 10, 11]) are possible as well. We systematically computed all Nash equilibria, at varying leakiness levels, for 189 amino acid pairs, corresponding to all possible pairs of 20 amino acids (Fig. 4b) except for one, namely the alanine and isoleucine pair, since the 00 genotype for this particular pair is also auxotrophic for a third amino acid (valine). Examples of higher-order interaction equilibria for a number of selected pairs are also provided in Supplementary Figs. 2–7.

As shown in Fig. 4b, a wide spectrum of equilibria ensues across different amino acid pairs and across different leakiness levels (e.g., see Fig. 4c, d). Interestingly, despite the diversity of exchanged metabolites and corresponding auxotrophic strains, a majority of pairs are found to conform to general ecological patterns: for example, 139 (out of 189 or 73.5%) of amino acid pairs display a qualitatively identical region of leakiness levels (green regions in Fig. 4b and c), where a unidirectional dependency, [00, 11], and cross-feeding, [01, 10], both emerge as Nash equilibria. The leakiness levels leading to these two equilibria are the ones for which the full producer (i.e., 11 genotype) can still sustain growth. We refer to this region as “sustainable leakiness region” (see “Methods” for further details). This is analogous to the maximum leakiness level in the study of individual amino acid secretions (Fig. 3b): we expect the leakiness of the two amino acids by *E*. *coli* in natural environments to lie in this region, as any leakiness levels outside this region would lead to extinction of the WT.

Another feature common to several amino acid pairs in Fig. 4b is the existence of a region in the leakiness plane (shown in red), where cross-feeding (i.e., [01, 10]) is the only viable association; excessive leakiness in this region makes the full producer, 11, non-viable (e.g., see Fig. 4c). Notice that this region is contiguous to the green region (i.e., the sustainable leakiness region with [00, 11] and [01, 10] as Nash equilibria). One interesting aspect of this configuration is that cross-feeding could initially ensue in the green region and gradually move toward the red region as leakiness levels of the two amino acids increase. This transition could be achieved in two phases: first, an initial cross-feeding association due to natural leakage of the amino acids (in the green region) could be spontaneously established; in a second phase, i.e., in a period of adaptive evolution, amino acids leakage by cross-feeders could increase to help the growth of the partner in exchange for the increased availability of metabolites each needs. The leakiness can increase to levels that cannot be sustained by their ancestor, 11, thereby providing a selective advantage for cross-feeders and ultimately leading to cooperative cross-feeding as the only viable association.

### Metabolic pathway interactions shape ecological interactions

In addition to exploring the landscape of Nash equilibria across different leakiness levels, it is interesting to ask whether the details of the biochemical networks underlying the genome-scale metabolic model predictions matter, and whether they provide direct explanatory power at the ecological level. Notably, for 50 amino acid pairs, the region of sustainable leakiness levels does not conform to the general pattern described above. For some pairs, this region is partitioned into a number of subregions each corresponding to a different equilibrium (e.g., see Fig. 4d), while in some extreme cases, this entire region corresponds to one type of Nash equilibrium, e.g., for arginine and glutamate, and glycine and threonine (see below).

**Fig. 5:**
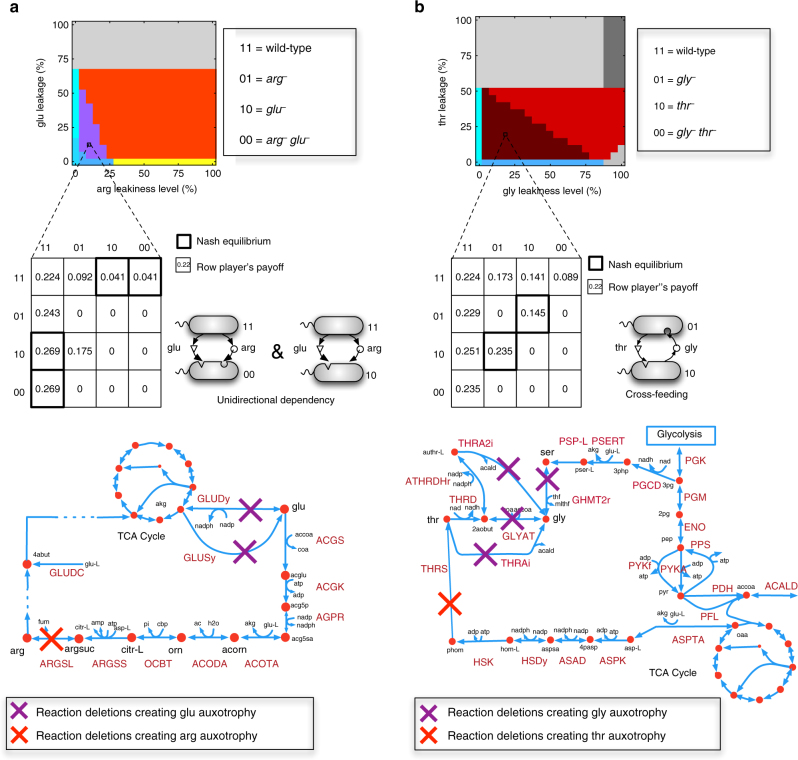
Metabolic pathway interactions can shape inter-species metabolic dependencies. The identified Nash equilibria for **a** arginine and glutamate and **b** glycine and threonine. Sample payoff matrices are given for the region of sustainable leakiness levels by 11 genotypes. Unidirectional dependency is the only possible equilibrium in this region for (arginine, glutamate) while cross-feeding is the only possible equilibrium for glycine and threonine. Metabolic maps show the metabolic pathways involved in the biosynthesis of the respective amino acids

For the first anomalous pair, (arginine and glutamate), Nash equilibria in the sustainable leakiness region includes [10, 11] and [00, 11], but not the cross-feeding state [01, 10] (Fig. 5a). Inspection of the biosynthesis pathway of arginine and glutamate in *E*. *coli* revealed that glutamate is required for the production of ornithine, which serves as an essential precursor for the biosynthesis of arginine. This implies that a mutant strain lacking the biosynthesis pathways for glutamate (i.e., strain 10) will not be able to synthesize and leak arginine, thus acting like a 00 genotype and preventing the occurrence of cross-feeding. This observation is consistent with a previous study reporting the inability of arginine and glutamate auxotrophic mutant strains to grow in a co-culture under minimal medium^3^. This effect is thus due to a pleiotropic metabolic gene (i.e., a gene whose modification affects more than one metabolic phenotype), and illustrates how core biochemistry can impact ecological interactions.

A more complex scenario occurs for the glycine and threonine pair, where cross-feeding, [01, 10], is the only Nash equilibrium that emerges in the sustainable leakiness region (Fig. 5b). What prevents [00, 11] from being a Nash equilibrium here? One of the conditions for [00, 11] to be a Nash equilibrium is that, in the presence of 11, the fitness of the non-producer (00) should be higher than any of the partial producers (01 and 10) (as one would intuitively expect, because 00 does not incur the production cost of the two amino acids). In this case, however, it turns out that 00 is less fit than 10 (*thr* ^−^ mutant) when facing 11, thereby preventing [00, 11] from being a Nash equilibrium. This anomalous effect is due to the fact that the concurrent removal of glycine and threonine biosynthesis genes (in 00) will lead to a reduction in the capacity of metabolic network to produce a number of other essential biomass components (such as serine). Interestingly, this is a case of diminishing-return (or “negative”) epistasis, in which the effect of the double mutation (00) is less severe than expected based on the two single mutations (01 and 10). Negative epistatic interactions preventing the appearance of [00, 11] as the Nash equilibrium can be observed for 34 other amino acid pairs (Supplementary Data 3). This pattern is consistent with existing experimental reports showing that epistasis correlates negatively with the expected fitness of multiple “genome streamlining” mutations in *E*. *coli*, thereby causing diminishing returns^40^. These results provide mechanistic insights into how epistatic interactions among intracellular pathways can affect ecological networks, a feature that cannot be easily captured by abstract phenomenological models.

### Paths toward the emergence of cross-feeding

**Fig. 6:**
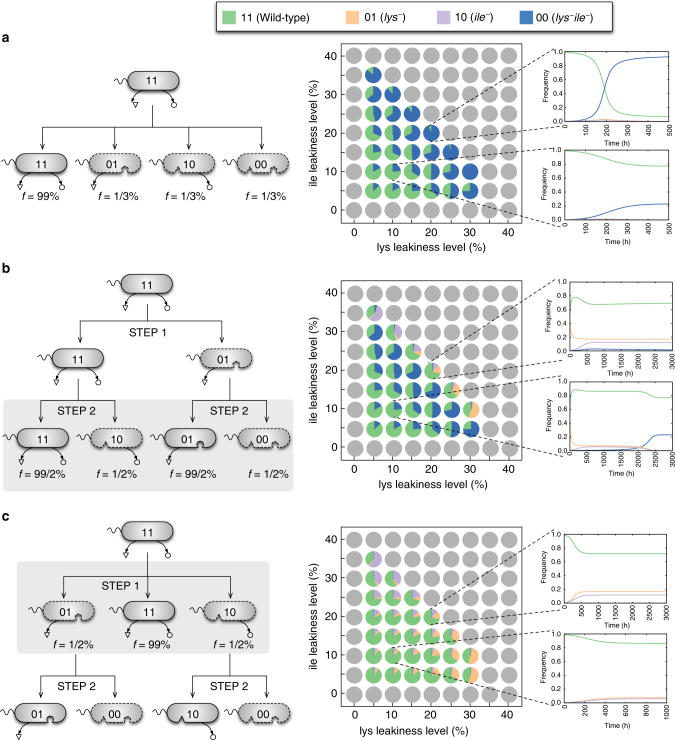
Impact of the initial genotype frequencies on the evolutionary emergence of metabolic dependencies in populations of *E*. *coli* with two leaky amino acids. Here, we have shown the results of targeted in silico invasion experiments for a representative amino acid pair (lysine and isoleucine) (see also Fig. 4c). **a** 00, 01, and 10 simultaneously originate from 11 (i.e., WT) through genome streamlining and invade an existing population of 11 genotypes. **b** A small population of 10 and 00 invade a resident population of the 11 and 01. This simulates the second step of a two-step process for the loss of the leaky functions hypothesized in ref. ^10^ (see the main text for details and also Supplementary Fig. 8). **c** A small population of 01 and 10 invades a resident population of 11. This models an alternative scenario for the two-step loss of leaky functions leading to stable cross-feeders: Two partial producer mutant genotypes (01 and 10) simultaneously originate from 11, followed by the rise of the 00 genotype from 01 and/or 10 in a later stage. As shown here, cross-feeders can evolutionarily stabilize and coexist with 11 genotypes in the first step. Further analysis showed that cross-feeders are also resistant to invasion by 00 genotypes arising in the second step (see Supplementary Fig. 9). Dynamic plots in **a**–**c** show the sample evolutionary dynamics of the system for selected equal leakiness levels for both lysine and isoleucine. Pie charts show the equilibrium frequencies of each genotype starting from the initial genotype frequencies shown in each panel. These equilibrium frequencies are given only for the sustainable leakiness region (green region in Fig. 4c)

It is next interesting to ask whether our approach can shed light onto the possible paths toward the rise of different interactions, especially cross-feeding. In the landscapes of identified Nash equilibria (Fig. 4b), cross-feeding ([01, 10]) emerges together with other equilibria (such as [00, 11] in the green region in Fig. 4b, c). This raises the question of whether and under what conditions a cross-feeding dependency would be evolutionarily stable. By performing a number of targeted in silico invasion experiments (see “Methods”), we found that this depends strongly on the initial frequencies of the four genotypes shown in Fig. 4a. In particular, our analysis demonstrates that cross-feeders (01 and 10) will go extinct if they invade the full producer (11) in presence of the non-producer genotype (00) (Fig. 6a). However, cross-feeders can subsist in a progressive loss of prototrophy, where 00 is not present initially and invades at a later stage. Figure 6b, c shows two examples of such scenario. For instance, the left diagram in Fig. 6b depicts the two-step process suggested in ref. ^10^: first, the biosynthetic capacity for one amino acid is lost, e.g., resulting in a 01 genotype, which could equilibrate and coexist with 11 (as shown in Fig. 3a, b, d). In the second step, either 01 or 11 may lose their capacity to produce the second amino acid (because the other strain can compensate), giving rise to 00 and 10 genotypes, respectively (Fig. 6b). Here, we quantitatively explored this mechanism by assessing the evolutionary dynamics for the second step, assuming that equilibration of the first step has already occurred, i.e., we performed in silico invasion experiments where 10 and 00 invade a resident population of 11 and 01. As shown in Fig. 6b, the prototrophic (11) and no-producer (00) genotypes always survive in this competition, while cross-feeders survive only at high leakiness levels. Consistent with our findings in Fig. 6b, c, a previous study showed that a heterogeneous community of *S*. *cerevisiae* auxotroph strains relying on each other for the exchange of amino acids can emerge from an initial prototrophic strain through the progressive loss of amino acids synthetic capacity^41^. Further in silico invasion experiments demonstrated that established cross-feeding pairs tend to be resistant to invasion by non-producers (Supplementary Fig. 9) and by prototrophs (Supplementary Fig. 10). Thus, once established, obligate mutual metabolic exchange could be evolutionarily stable against invasion by other genotypes, even in a homogenous environment, consistent with previous experimental reports^42^. In addition, as evident from Fig. 6, whether the division of labor afforded by mutual metabolic exchanges can lead to the establishment of a cross-feeding association depends on the initial genotype frequencies, the level of metabolic exchanges (leakiness) and the nature of the specific metabolites exchanged.

## Discussion

We demonstrated here that by adding new layers of details to abstract theoretical ecology models, we can reveal how intracellular molecular mechanisms (including pleiotropy and epistasis of metabolic genes) lead to the rise of non-intuitive ecological interactions. The analysis we presented spans over 80,000 in silico experiments (across 189 amino acid pairs and 441 leakiness level combinations), which is beyond the current experimental capabilities. This study provides testable predictions that can be used as a guideline for the design of future-targeted experiments, e.g., built upon previously established synthetic communities^3–7^. For example, our computational results could be used to suggest choices of metabolite pairs and ranges of leakiness levels likely to lead to the establishment of a specific inter-dependency, such as, cross-feeding. Targeted experiments based on our computational results could further help assess the BQ Hypothesis. In particular, one could explicitly test the feasibility of different paths toward the establishment of cross-feeding mediated by the presence of BQ functions, as shown in Fig. 6. Future theoretical developments built upon this work could examine how the rise of obligate metabolic dependencies based on BQ functions may serve as a starting point for the evolution of cooperative behavior, e.g., through the evolution of upregulated leakiness levels (as described for the transition from the green to the red region in Fig. 4c). One limitation of the current study is that, due to the use of the Replicator equation for our in silico invasion experiments we do not take into account the impact of population dynamics (changes in population size) on evolutionary dynamics (changes in genotype frequencies)—a phenomenon referred to as eco-evolutionary feedback^43–46^. This interplay may affect the equilibria of the system^44–46^, potentially causing our in silico predictions (Fig. 6) to deviate from experimental observations. Future studies could address these possible discrepancies by extending our approach to eco-evolutionary game theory models. Alternatively, dynamic flux balance analysis (FBA) of microbial consortia^24^ could be used to capture both eco-evolutionary feedback as well as the impact of dynamic changes of the growth medium.

From a biotechnological standpoint, our study offers a basis for better understanding metabolic interdependencies in biomedically relevant natural microbial communities, such as those in the human gut microbiota^47–49^: here products from the bacterial degradation of ingested food (e.g., starch by *Bacteroidetes* ^47^) can serve as public goods for the microbiota, potentially leading to the rise of cheaters, with major consequences for the health of the human host. In addition, our approach lays the foundation for proactively incorporating evolutionary concepts in the de novo design of stable synthetic microbial consortia for various biotechnological applications, guaranteeing that engineered communities will be resistant to invasion by competing strategies^31^.

## Methods

### Background on evolutionary game theory

Evolutionary game theory is the application of classical game theory to model the evolutionary dynamics of mixed populations. Modeling microbial communities with evolutionary game theory involves two steps: (i) Considering all pairwise interactions among genotypes and estimating the payoff (fitness) of a microbial player *k* upon interacting with a partner *k*′. Higher-order interactions can be similarly considered. These estimated payoffs are represented in the form of a matrix, referred to as the payoff matrix of the game. (ii) Using the payoff matrix to identify the Nash equilibria—a fundamental concept in game theory, defined as a state where no player has an incentive to unilaterally change its current strategy, because it cannot improve its payoff by doing so. An “evolutionary stable strategy” is a similar concept in evolutionary game theory: It is a Nash equilibrium, which is evolutionarily stable, i.e., natural selection alone is sufficient to prevent invasion by competing mutant strategies. Evolutionary stable strategies can be found by modeling the evolutionary dynamics of the game using the computed payoffs (see the following sections). In this text (following evolutionary game theory^33^ and microbial ecology literature^44^), by “evolutionary dynamics,” we mean how the relative genotype abundances (frequencies or community structure) change over time. This aspect of evolutionary dynamics, as opposed to the simulation of de novo mutations and subsequent selection processes (which have been pursued in other studies^50^), is the main focus of our analyses.

### Background on Flux Balance Analysis (FBA)

FBA (described in detail elsewhere, e.g., ref. ^51^) is a linear optimization problem that uses genome-scale metabolic models to make quantitative predictions about the cell’s growth capacity, intracellular reaction fluxes, and secretion rates of metabolites that are potentially excreted by the cell under a given environmental/growth condition. In the most common formulation, this is achieved by maximizing the flux of a pseudo-reaction called biomass reaction (*v*_biomass_) whose reactants are precursors required for growth and whose flux is indicative of the cell’s growth capacity. This is subject to constraints imposing steady-state mass balance for each metabolite in the network (see Constraint 1 below) and lower and upper bounds on reactions fluxes reflecting known irreversibility of specific reactions and uptake and aeration conditions (see Constraint 2 below). The standard formulation of FBA is as follows:

1 $$\begin{array}{*{20}{l}}\\ {\begin{array}{*{20}{l}}\\ {{\rm{Maximize}}\,{v_{{\rm{biomass}}}}} \hfill \\ \\ {{\rm{subject}}\,{\rm{to}}} \hfill \\ \end{array}} \hfill & {} \hfill \\ \\ {\mathop {\sum}\limits_{j \in J} {{s_{ij}}{v_j}} = 0,} \hfill & {\forall i \in I,} \hfill \\ \end{array}$$

2 $$\begin{array}{*{20}{l}}\\ {{\rm{L}}{{\rm{B}}_j} \le {v_j} \le {\rm{U}}{{\rm{B}}_j},} \hfill & {\forall j \in J,} \hfill \\ \end{array}$$

where, *I* is the set of metabolites, *J* is the set of reactions, *s*_*ij*_ is the stoichiometric coefficient of metabolite *i* in reaction *j* (known from the metabolic model), LB_*j*_ and UB_*j*_ denote lower and upper bounds on the flux of reaction *j*, respectively, and *v*_*j*_ is the flux of a reaction *j*.

### Using FBA to compute payoffs of interacting microbes

We used FBA to provide organism-specific estimates of the payoffs upon specific pairwise (or higher-order) interactions (see also Fig. 1 and Supplementary Figs. 11 and 12). For a given pair of genotypes *k* and *k*′, we solve two separate FBA problems, one for the genome-scale metabolic model associated with genotype *k* and one for the genome-scale metabolic model associated with genotype *k*′. The optimal biomass flux obtained upon solving these FBA problems will provide an estimate of the growth rate of each genotype in a given pairwise interaction, which we use as a proxy for its payoff. For example, the payoff of *k* when facing *k*′ (*a*_*kk*′_) is $v_{{\rm{biomass}}}^k$ and the payoff of *k*′ when facing *k* (*a*_*k*′*k*_) is $v_{{\rm{biomass}}}^{k\prime }$.

Each FBA problem involves two new types of constraints in addition to Constraints (1) and (2) mentioned above: (i) The first type of constraint (Eq. 3 below) implements in silico the gene deletions that correspond to the genotype under consideration. For example, if genotype *k* is auxotroph for lysine, this auxotrophy can be induced by knocking out gene *lysA*, which codes for diaminopimelate decarboxylase (DAPDC). In the FBA model, this gene deletion is simulated by setting *v*_DAPDC_ = 0. More complex gene-to-reaction mappings could lead to more complex set of constraints. In cases where several different choices are possible for genes whose deletion would induce a given auxotrophy, we select one arbitrarily. (ii) The second type of constraints (Eqs. 4 and 5 below) simulates the exchange of metabolites between the genotypes under consideration. Given the rate (*e*_*i*_) at which a metabolite *i* leaks out of genotype *k*, we simulate such leakage by imposing a fixed secretion rate *e*_*i*_ in the FBA calculation for this genotype. In simulating growth for genotype *k*, we further take into account the availability of other metabolites secreted by genotype *k*′, by appropriately setting the import constraints in the FBA problem. Thus, in our approximation of an interaction, the two FBA calculations for genotypes *k* and *k*′ are performed independently, each assuming availability of the metabolites leaked by the other.

The general FBA problem for genotype *k* is mathematically formulated as follows (superscript *k* is removed here for the ease of presentation):

$$\begin{array}{*{20}{l}}\\ {\begin{array}{*{20}{l}}\\ {{\rm{Maximize}}\,{v_{{\rm{biomass}}}}} \hfill \\ \\ {{\rm{subject}}\,{\rm{to}}} \hfill \\ \end{array}} \hfill & {} \hfill \\ \\ {\mathop {\sum }\limits_{j \in J} {\kern 1pt} {s_{ij}}{v_j} = 0,} \hfill & {\forall i \in I,} \hfill \\ \\ {{\rm{L}}{{\rm{B}}_j} \le {v_j} \le {\rm{U}}{{\rm{B}}_j},} \hfill & {\forall j \in J,} \hfill \\ \end{array}$$

3 $$\begin{array}{*{20}{l}}\\ {{v_j} = 0,} \hfill & {\forall j \in {J^{{\rm{mutation}}}}} \hfill \\ \end{array}$$

4 $$\begin{array}{*{20}{l}}\\ {{v_{{\rm{EX}}\_i(e)}} \ge {e_i},} \hfill & {\forall i \in {I^{{\rm{leaky}}}}} \hfill \\ \end{array}$$

5 $$\begin{array}{*{20}{l}}\\ {{v_{{\rm{EX}}\_i(e)}} \ge - {u_i},} \hfill & {\forall i \in {I^{{\rm{uptake}}}}} \hfill \\ \end{array}$$

where, *J* ^mutation^ denotes the set of reactions corresponding to specific gene mutations for the genotype under consideration. In addition, *I* ^leaky^⊂ *I* is the set of leaky (secreted) metabolites by the genotype under consideration and *I* ^uptake^⊂ *I* represents the set of metabolites that is available to this genotype for uptake and that is provided by other genotypes. *v*_EX_*i*(*e*)_ denotes the flux of exchange reaction for a metabolite *i*. *e*_*i*_ > 0 and *u*_*i*_ > 0 denote the pre-specified net export and uptake flux of a metabolite *i*, respectively (see Supplementary Methods for details of how *e*_*i*_ and *u*_*i*_ were calculated for the presented case studies).

In the above FBA problem, Constraint (3) sets to zero the flux of reactions corresponding to the specific gene mutations in the genotype under consideration. Constraint (4) requires the export of leaky metabolites at the pre-specified level *e*_*i*_ and Constraint (5) allows for the uptake of metabolites available from partner genotype(s) in a pairwise or (higher-order) interaction. It is worth highlighting again that the input parameters for this FBA problem (in addition the metabolic network stoichiometry) are the list of metabolites that each genotype is leaking, and the level of leakiness for each of them.

The payoff of the genotype under consideration is set to the optimal value of the biomass flux, or to the death rate (a negative value) in the case of an infeasible problem. An infeasible FBA problem may occur due the lack of enough carbon source to satisfy maintenance ATP requirements in the model or due to imposing a high level of leakiness for leaked metabolites. Imposed leakiness level causing this infeasibility is referred to “unsustainable leakiness levels.” The details of specific formulations for the presented case studies with *S*. *cerevisiae* and *E*. *coli* are given in the Supplementary Methods. Additional environmental/strategic/genetic conditions can be incorporated through the addition of appropriately defined constraints. Alternatively, one can use a different objective function (e.g., the minimization of metabolic adjustment^52^), objective function-independent approaches^53, 54^, or other constraint-based community modeling tools, e.g., those in refs. ^21,23^.

### Automated identification of the Nash equilibria

Upon constructing the payoff matrix, as described above, one can identify the Nash equilibria of the game. We developed NashEq Finder, an optimization-based procedure to automate the identification of all pure strategy Nash equilibria of an *n*-player game. Here, for ease of presentation, we describe the NashEq Finder formulation for a two-player non-symmetric game. Let *P* and *Q* denote the set of all conceivable strategies for player 1 and player 2, respectively. A binary decision variable is defined as follows to capture whether or not each entry *pq* of the payoff matrix satisfies the conditions of a Nash equilibrium:

$${{w_{pq}} = \left[ {\begin{array}{*{20}{l}} {1,} \hfill & {{\rm{If}}\,{\rm{entry}}\,pq\,{\rm{satisfies}}\,{\rm{the}}\,{\rm{conditions}}\,{\rm{of}}\,{\rm{a}}\,{\rm{Nash}}\,{\rm{equilibrium}}} \hfill \\ {0,} \hfill & {{\rm{Otherwise}}} \hfill \\ \end{array}} \right.,\,\forall p \in P,\,q \in Q.}$$

Now, let the entry *pq* of the payoff matrix constitute $\left( {a_{pq}^1,a_{pq}^2} \right)$with $a_{pq}^1$ and $a_{pq}^2$ being the payoffs of players 1 and 2, respectively. NashEq Finder can be formulated as follows:

$$\begin{array}{*{20}{l}}\\ {{\rm{Maximize}}\;z = \mathop {\sum}\limits_{p \in P} {\mathop {\sum}\limits_{q \in Q} {{w_{pq}}} } } \hfill & {\left[ {{\rm{NashEq}}\,{\rm{Finder}}} \right]} \hfill \\ \\ {{\rm{subject}}\,{\rm{to}}} \hfill & {} \hfill \\ \end{array}$$

6 $$a_{pq}^1 \ge \left( {\mathop {{\rm{max}}}\limits_{p\prime \in P} \left\{ {a_{p\prime q}^1} \right\}} \right){w_{pq}} + {\rm{L}}{{\rm{B}}_1}\left( {1 - {w_{pq}}} \right)\;\forall p \in P,q \in Q,$$

7 $$a_{pq}^2 \ge \left( {\mathop {{\rm{max}}}\limits_{q\prime \in Q} \left\{ {a_{pq\prime }^2} \right\}} \right){w_{pq}} + {\rm{L}}{{\rm{B}}_2}\left( {1 - {w_{pq}}} \right)\;\forall p \in P,q \in Q,$$

where, LB_1_ and LB_2_ are non-zero lower bounds on the payoff values of players 1 and 2, respectively, The objective function of this optimization problem maximizes the values of binary variables corresponding to entries of the payoff matrix. The definition of binary variable *w*_*pq*_ is mathematically imposed by Constraints (6) and (7). In particular, Constraint (6) mathematically describes the conditions of a pure strategy Nash equilibrium for player 1, i.e., if player 2’s strategy is fixed at *q* ∈ *Q*, player 1 attains its maximum payoff by taking strategy *p* ∈ *P*. Constraint (7) imposes the same condition for player 2 (see Supplementary Methods for an example detailing how these constraints work). Notice that NashEq Finder is an integer linear program (ILP), which can be always solved to global optimality (if an optimal solution exists). Upon solving this optimization problem, any entry of the payoff matrix for which the corresponding binary variable is equal to one will be a Nash equilibrium. NashEq Finder can thus identify all pure strategy Nash equilibria of the game in one shot by solving this ILP problem. If this optimization problem is infeasible, it means that no pure strategy Nash equilibrium exists. This formulation can be easily generalized for an *n*-player game. A python script implementing NashEq Finder for an *n*-player game is available in Supplementary Software 1. A rudimentary assessment of the computational efficiency of the NashEq Finder for the case studies presented in this paper is also provided in Supplementary Methods.

### Modeling evolutionary dynamics at genome-scale resolution

Following standard approaches in evolutionary game theory, we model evolutionary dynamics using the Replicator equation^33^. To take into account interactions higher than pairwise, we used an extended form of the classical Replicator equation^33^. This equation predicts the changes in the relative abundance (frequencies) of genotypes over time according to their reproductive fitness under the assumption of a roughly constant population size^33^ (see Supplementary Note 1 for the difference between this equation and multi-species dynamics FBA^24^). The extended form of the Replicator equation can be expressed as follows^55^:

8 $$\frac{{{\rm{d}}{x_k}}}{{{\rm{d}}t}} = \left( {{f_k}\left( {\boldsymbol{x}} \right) - \phi \left( {\boldsymbol{x}} \right)} \right){x_k}{\rm{,}}\;k = 1,2, \ldots ,K,$$

9 $${f_k}\left( {\boldsymbol{x}} \right) = \mathop {\sum}\limits_{k\prime = 1}^K {{a_{kk\prime }}{x_{k\prime }}} + \mathop {\sum}\limits_{k\prime = 1}^K {\mathop {\sum}\limits_{k{{''}} = 1}^K {{a_{k{k^{\prime}}{k^{''}}}}{x_{k'}}{x_{{k^{''}}}} + \ldots } } ,\;k = 1,2, \ldots ,K,$$

10 $$\phi \left( {\boldsymbol{x}} \right) = \mathop {\sum}\limits_{k\prime = 1}^K {{f_{k\prime }}\left( {\boldsymbol{x}} \right){x_{k\prime }}} .$$

Here, index *k* denotes a community member *k* and ***x*** = [*x*_1_, *x*_2_, …, *x*_*K*_]^*T*^ is the composition of the community with *x*_*k*_ being the frequency of genotype *k* at time *t*. *f*_*k*_(***x***) is the average reproductive fitness of genotype *k* that depends not just on other genotypes it may encounter but also on their frequencies. *a*_*kk*′_ and *a*_*kk*′*k*″_ denote the payoffs of genotype *k* when encountering another genotype *k*′ in a two-player game (i.e., pairwise interaction) or two other genotypes *k*′ and *k*″ in a three-player game, respectively. Finally, *ϕ*(***x***) is the average fitness of the entire community. Here, we use the Replicator equation to perform targeted in silico invasion experiments, in which a newly emerged low-frequency genotype invades an existing resident genotype.

### Code availability

A python script implementing NashEq Finder is available as Supplementary Software 1. In addition, source codes used to generate the data and figures analyzed during the current study are fully available from the corresponding author upon request.

### Data availability

The data sets generated and/or analyzed during the current study are available from the corresponding author on reasonable request.

## Electronic supplementary material

Supplementary Information

Description of Additional Supplementary Files

Supplementary Data 1

Supplementary Data 2

Supplementary Data 3

Supplementary Software 1
