# Supplementary material for: Genome-driven evolutionary game theory helps understand the rise of metabolic interdependencies in microbial communities
Source: Nat Commun. 2017 Nov 16;8:1563. doi: 10.1038/s41467-017-01407-5 (PMC5691134; doi:10.1038/s41467-017-01407-5)
Supplement: Supplementary file 1 — Supplementary Information [file 41467_2017_1407_MOESM1_ESM.pdf]

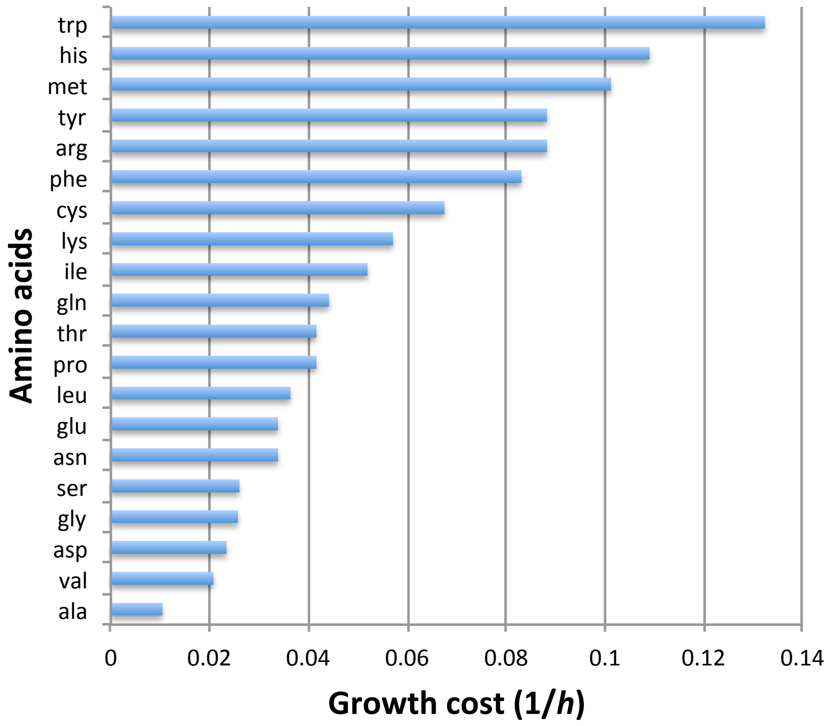

Supplementary Figure 1. Ranking of amino acids according to their *in silico* growth cost. The growth cost was computed as the reduction in growth rate of a wild-type strain upon requiring the model to produce 1 mmol/(gDW.h) of each amino acid.

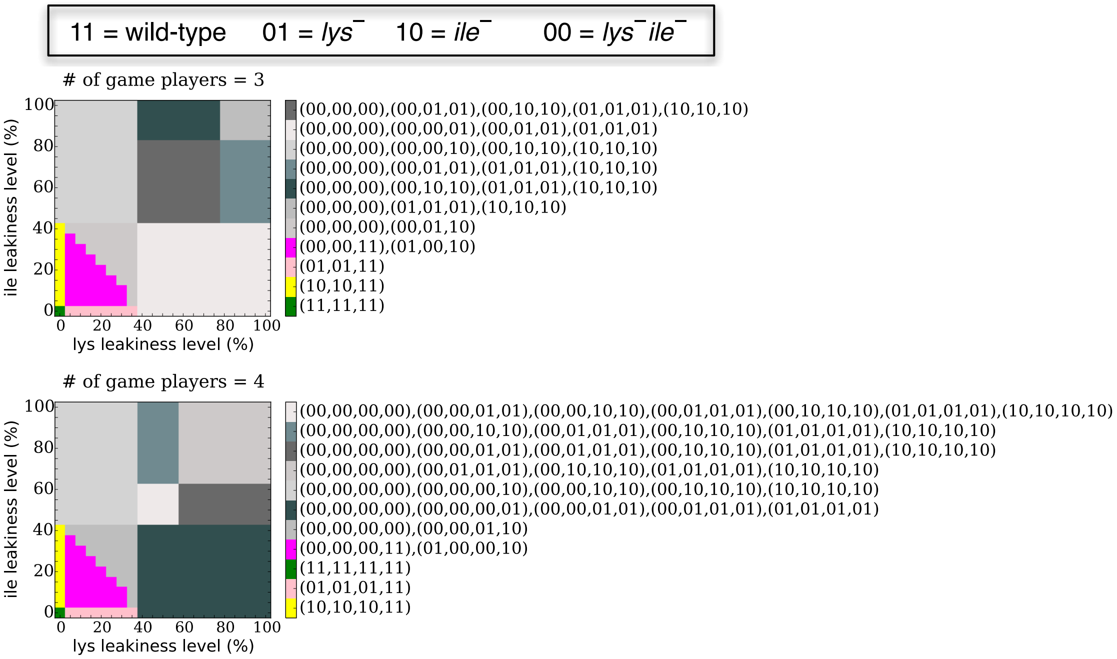

Supplementary Figure 2. All identified Nash equilibria (NEs) of three- and four-player games for (lysine, isoleucine) amino acid pair. Here, three- and four-player games mean the games involving the simultaneous encounter of three or four genotypes (as opposed to pairwise interactions) chosen from all possible genotypes (i.e., 11, 01, 10 and 00).

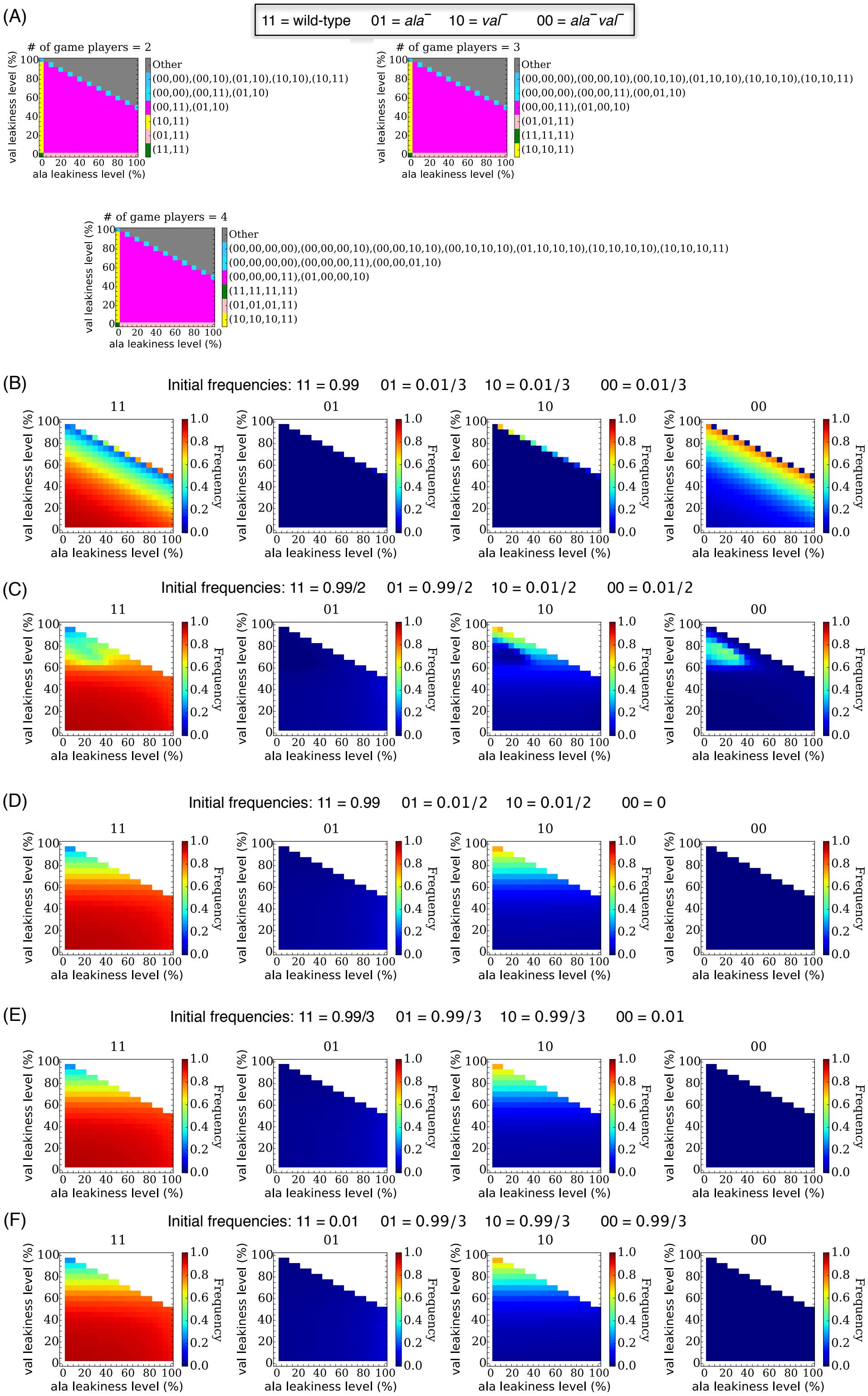

Supplementary Figure. 3. **Nash equilibria and equilibrium genotype frequencies for (alanine, valine) amino acid pairs.** Both alanine and valine are low-cost amino acids. (A) Identified Nash equilibria for two-, three- and four-player games. Equilibria labeled 'Other' represent the integration of all equilibria corresponding to leakiness levels that cannot be sustained by 11 genotype. (B) to (F) show the equilibrium frequencies of various genotypes (11, 01, 10 and 00) using different initial genotype frequencies each with direct biological interpretation (see the main text and Figure 6 therein).

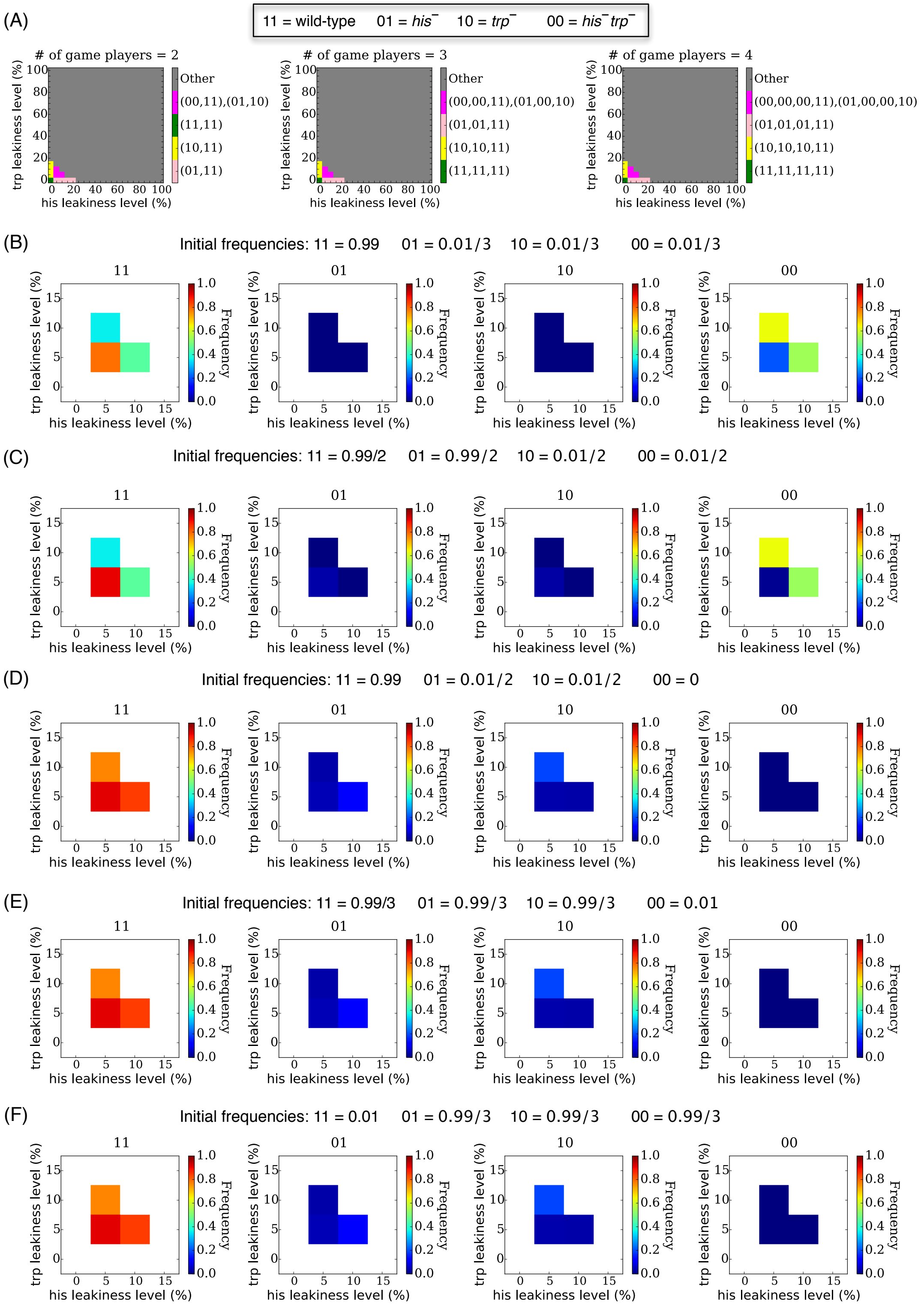

Supplementary Figure 4. **Nash equilibria and equilibrium genotype frequencies for (histidine, tryptophan) amino acid pairs.** Both histidine and tryptophan are high-cost amino acids. (A) Identified Nash equilibria for two-, three- and four-player games. Equilibria labeled 'Other' represent the integration of all equilibria corresponding to leakiness levels that cannot be sustained by 11 genotype. (B) to (F) show the equilibrium frequencies of various genotypes (11, 01, 10 and 00) using different initial genotype frequencies each with direct biological interpretation (see the main text and Figure 6 therein).

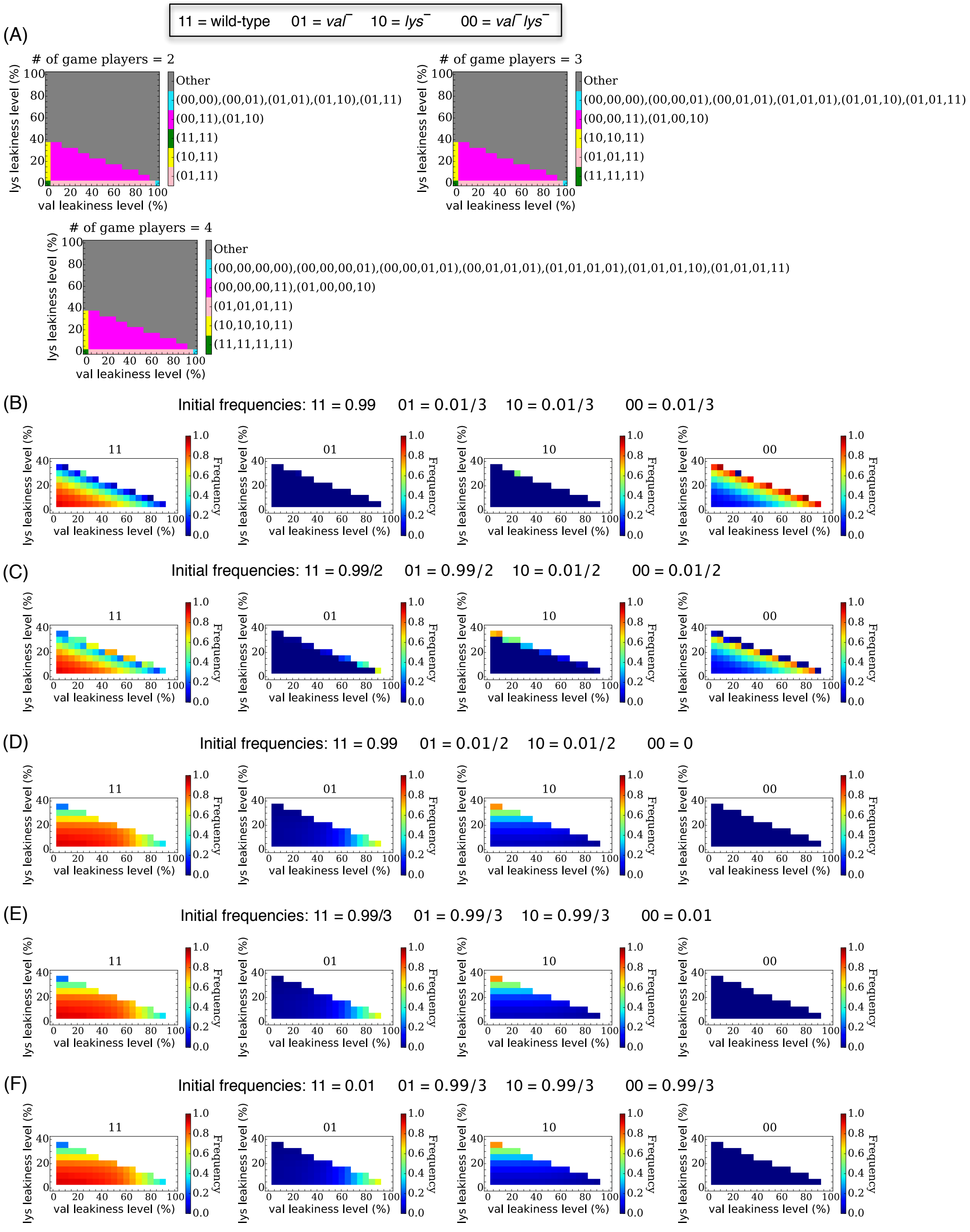

Supplementary Figure 5. **Nash equilibria and equilibrium genotype frequencies for (valine, lysine) amino acid pairs.** Valine and lysine are low- and medium-cost amino acids, respectively. (A) Identified Nash equilibria for two-, three- and four-player games. Equilibria labeled 'Other' represent the integration of all equilibria corresponding to leakiness levels that cannot be sustained by 11 genotype. (B) to (F) show the equilibrium frequencies of various genotypes (11, 01, 10 and 00) using different initial genotype frequencies each with direct biological interpretation (see the main text and Figure 6 therein).

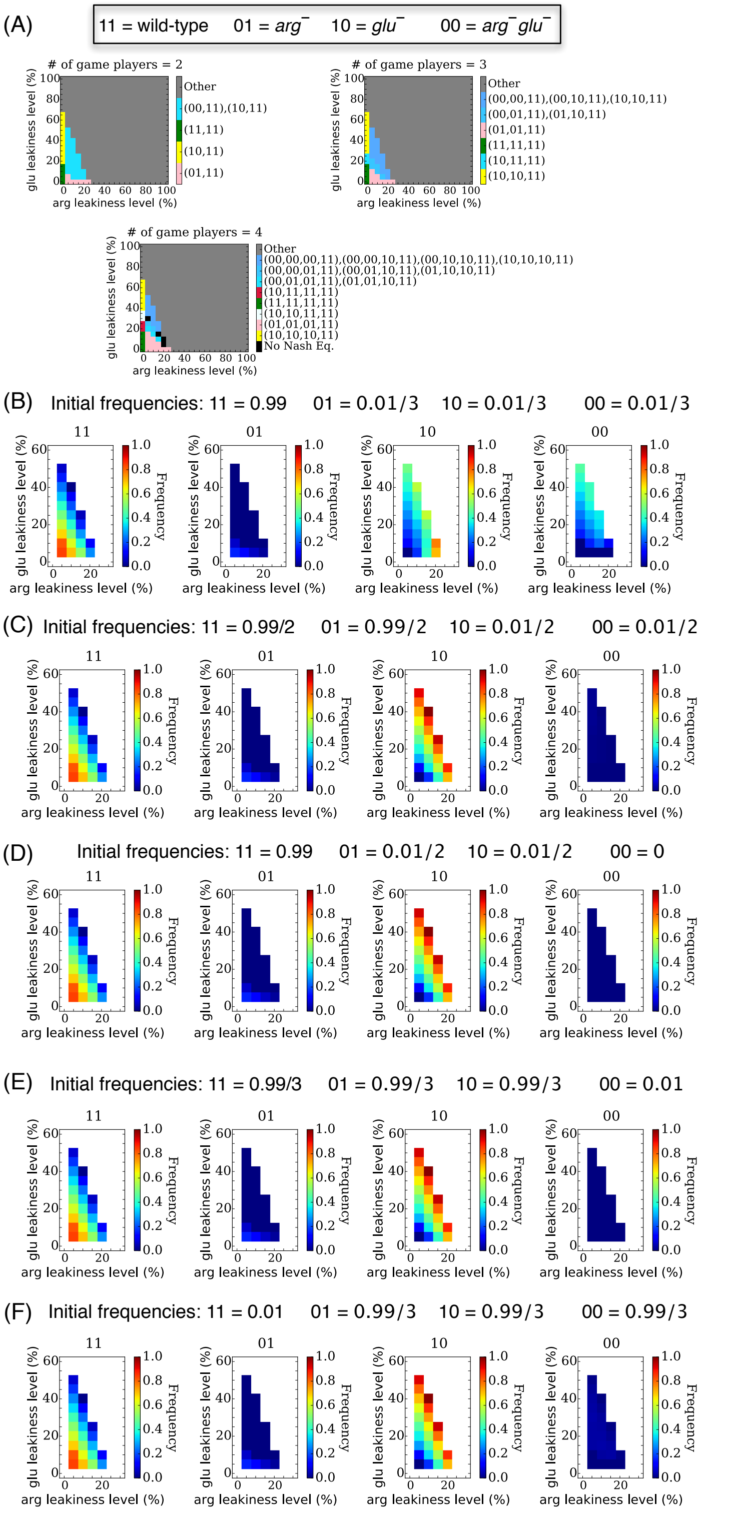

Supplementary Figure 6. **Nash equilibria and equilibrium genotype frequencies for (arginine, glutamate) amino acid pairs.** (A) Identified Nash equilibria for two-, three- and four-player games (see also Fig. 4A of the main text). Equilibria labeled 'Other' represent the integration of all equilibria corresponding to leakiness levels that cannot be sustained by 11 genotype. (B) to (F) show the equilibrium frequencies of various genotypes (11, 01, 10 and 00) using different initial genotype frequencies each with direct biological interpretation (see the main text and Figure 6 therein).

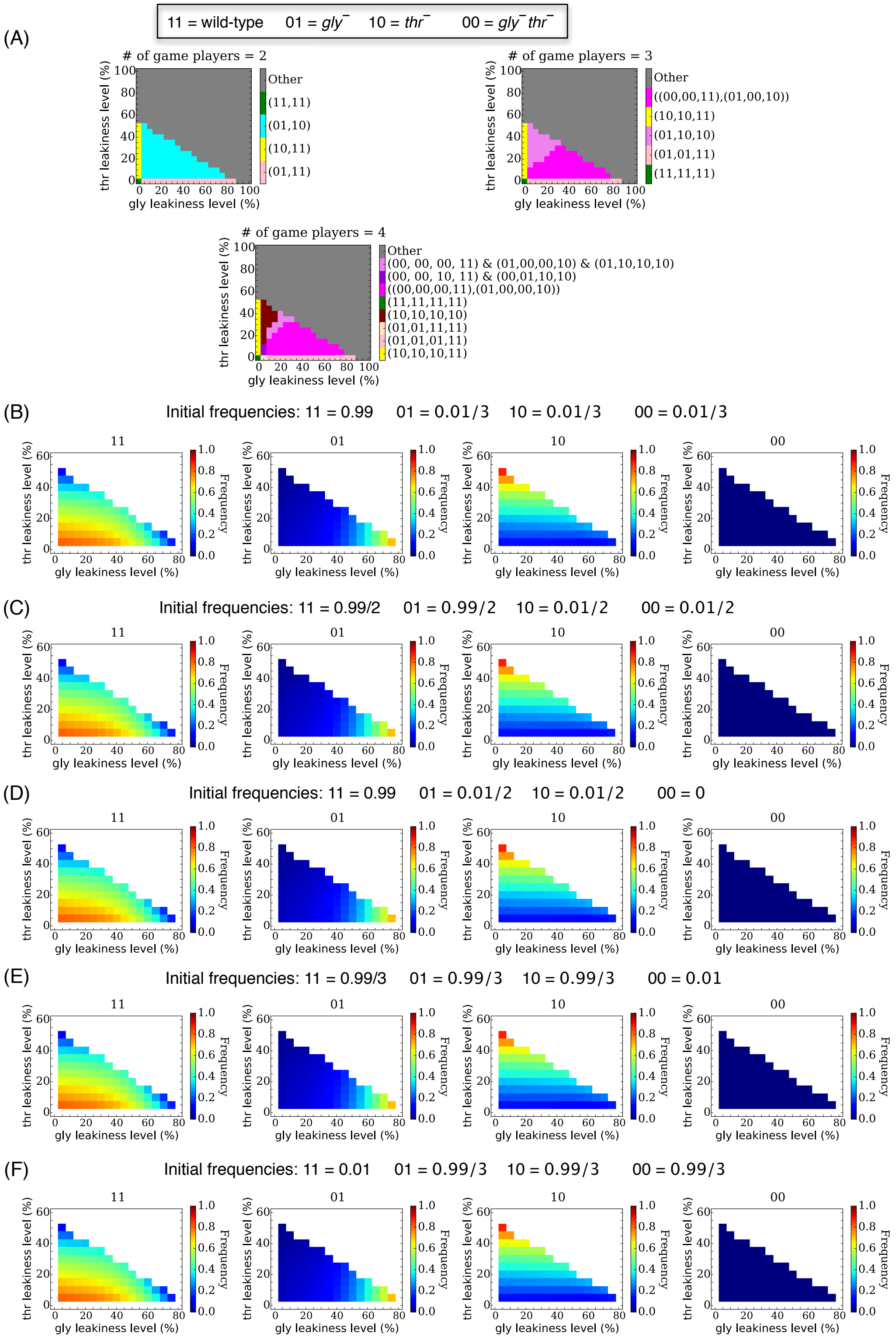

Supplementary Figure 7. **Nash equilibria and equilibrium genotype frequencies for (glycine, threonine) amino acid pairs.** (A) Identified Nash equilibria for two-, three- and four-player games (see also Figure 3F of the main text). Equilibria labeled 'Other' represent the integration of all equilibria corresponding to leakiness levels that cannot be sustained by 11 genotype. (B) to (F) show the equilibrium frequencies of various genotypes (11, 01, 10 and 00) using different initial genotype frequencies each with direct biological interpretation (see the main text and Figure 6 therein).

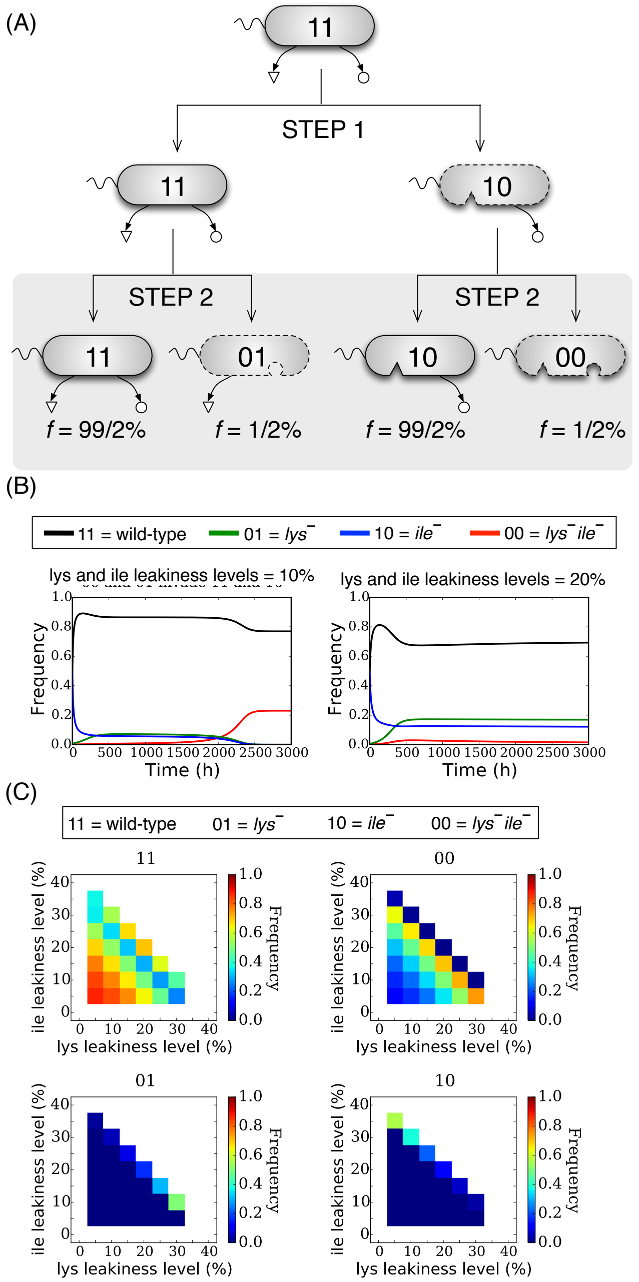

Supplementary Figure 8. **Equilibrium genotype frequencies in a two-step loss of the leaky functions does not depend on the order of losing functions.** (A) A small population of 00 and 01 genotypes invades an incumbent population of 11 and 10 genotypes (see also the main text and Figure 6B therein): 11 genotype is first converted to 10 by losing its second leaky function. Upon equilibrating, both 10 and 11 may lose their first leaky function converting to 00 and 01. (B) The frequency profiles of various genotypes for two representative low (10%) and high (20%) leakiness levels for both lysine and isoleucine. (C) Equilibrium frequencies of various genotypes for leakiness levels sustainable by 11 genotype (green region in Figure 4C of the main text). A comparison with Figure 6B of the main text shows that the equilibrium frequencies of the four genotypes do not depend on which leaky function is lost first. Assessing the evolutionary dynamics at low/moderate in (B) leakiness levels shows that even though cross-feeding emerges initially, it is eventually driven to extinction by non-producers. This pattern reveals an evolutionary path toward cross-feeding: Once a cross-feeding association is established at low/moderate leakiness levels, cross-feeders may evolve to increase their level of secreted amino acids to help their partner's growth in exchange for the amino acid they need. This increase can be so severe that even prototrophs (11) are driven to extinction (i.e., moving from the green to red region in Figure 4C of the main text).

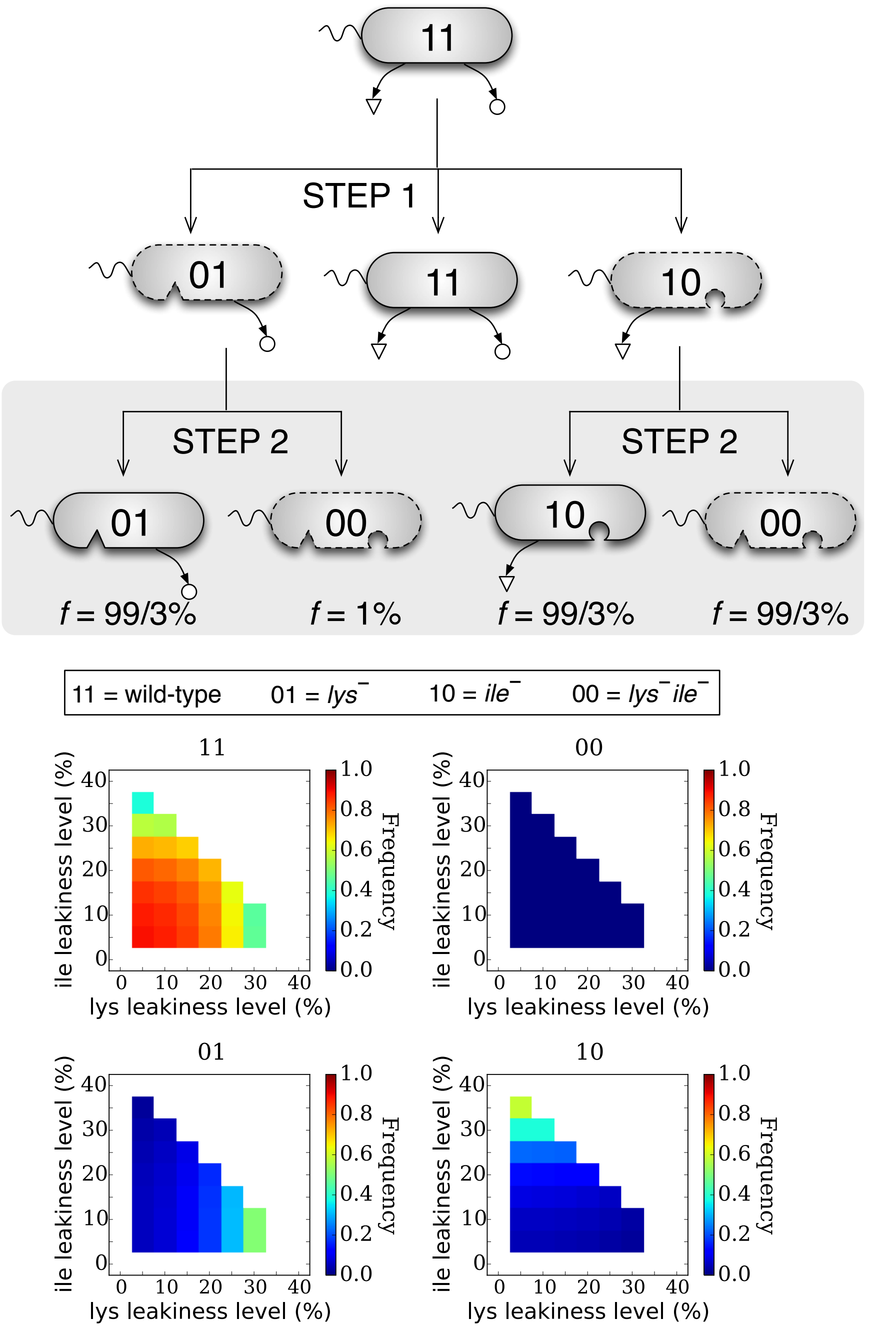

Supplementary Figure 9. **Cross-feeders are resistant to invasion by non-producers once established**. First, two mutant genotypes arise from 11, where one has lost its first leaky function (01) and the second has lost its second one (10). Next, 00 originates from 01 and/or 10 by losing their remaining leaky function. (A) and (B) show the initial and equilibrium genotypes frequencies for first step, respectively, and (C) and (D) show those for the second step for (lysine, isoleucine) amino acid pair. The equilibrium frequencies are shown only for leakiness levels that are sustainable by 11 genotype (green region in Figure 4C in the main text). These figures show that cross-feeding can emerge in the first step and can resist exploitation by 00 genotypes in the second step.

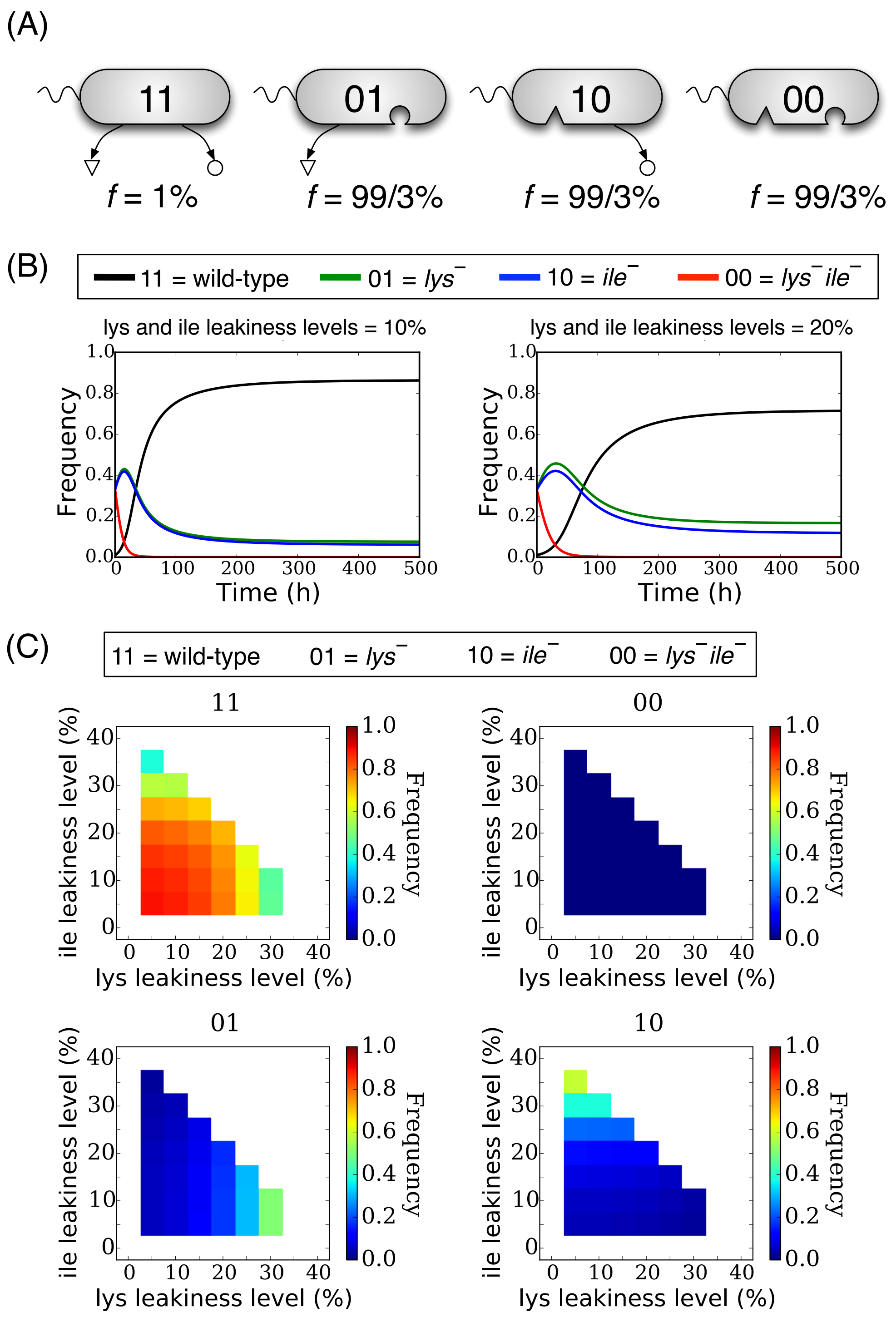

Supplementary Figure 10. Analysis of the outcome of a prototrophic genotype (11) invading a resident population of 00, 01 and 10 genotypes. Results are shown for (lysine, isoleucine) as a representative amino acid pair. (A) Initial genotype frequencies. (B) Genotype frequencies profiles for two representative low (10%) and high (20%) leakiness levels for both lysine and isoleucine. (C) Equilibrium genotype frequencies for leakiness levels sustainable by 11 genotype (green region in Figure 4C of the main text).

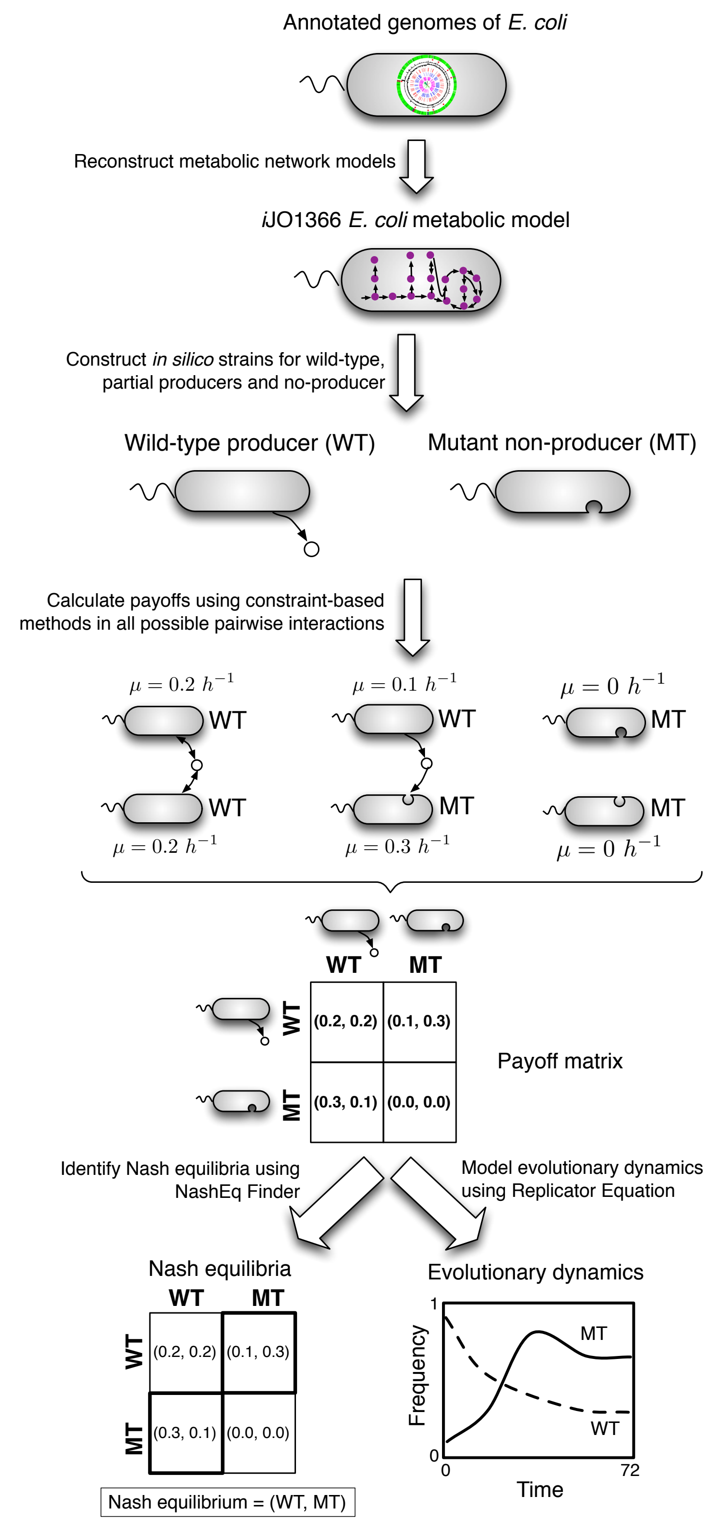

Supplementary Figure 11. A schematic representation of the metabolic network-driven evolutionary game theory approach used for *E. coli* leaking one amino acid. This same procedure can be applied to any system with one leaky trait such as sucrose hydrolysis by *S. cerevisiae* (see the main text).

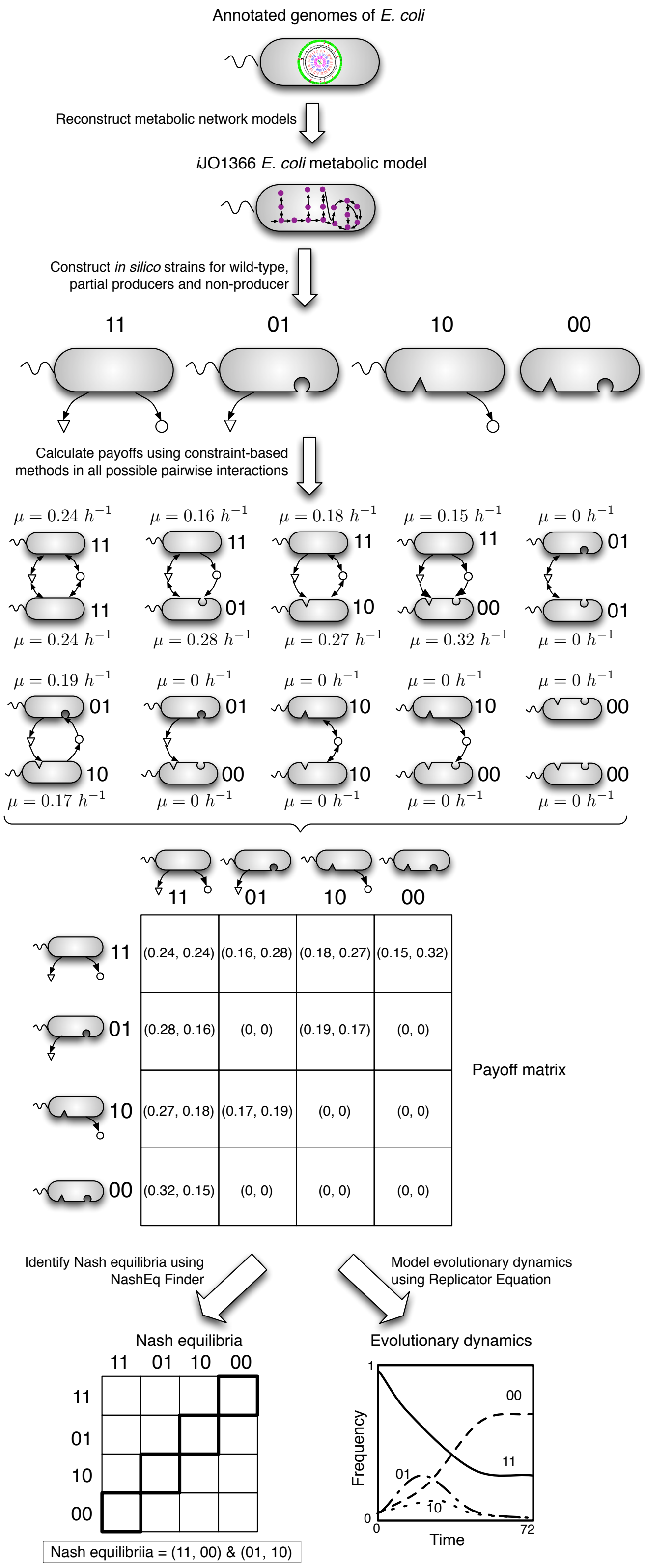

Supplementary Figure 12. A schematic representation of the metabolic network-driven evolutionary game theory approach used for *E. coli* leaking two amino acid. This same procedure can be applied to any system with two leaky traits.

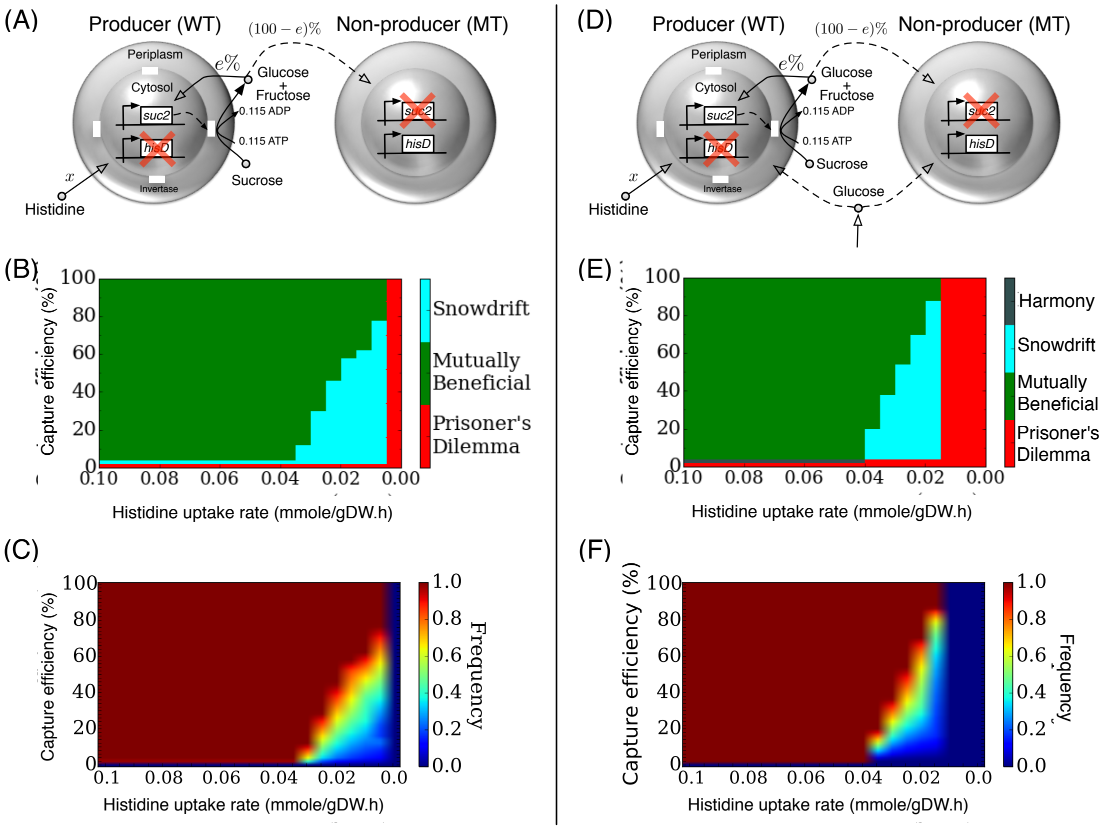

Supplementary Figure 13. **Metabolic inter-dependencies in invertase-producing *S. cerevisiae* system using a histidine auxotroph strain.** (A) Interactions between non-producer and histidine auxotroph producer strains of *S. cerevisiae*. and denote the histidine uptake rate and glucose/fructose capture efficiency, respectively. (B) Nash equilibria and (C) the equilibrium frequency of producers. Histidine uptake rate is shown in the reverse order on the horizontal axes as lowering the histidine uptake rate corresponds to increasing the cooperation cost. (D) Interactions between non-producer and histidine auxotroph producer strains of *S. cerevisiae* when additional glucose is provided in the growth medium. (E) Nash equilibria and (F) the equilibrium frequency of the producer in the presence of additional glucose in the growth medium. A Harmony game in (E) is a game where the optimal strategy is to take the same strategy as the opponent and corresponds to two Nash equilibria, namely, (Non-producer, Non-producer) and (Producer, Producer). Addition of glucose to the extracellular environment increases the average fitness of non-producers and makes producers more difficult to sustain regardless of how the cooperation cost is modeled.

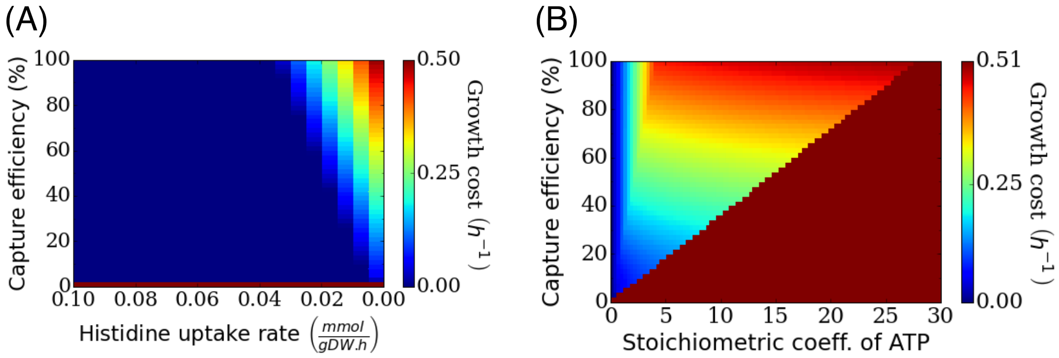

Supplementary Figure 14. **Sensitivity of predicted growth defect (cooperation cost) using the yeast iAZ900 model.** Sensitivities are shown with respect to (A) histidine uptake rate and (B) stoichiometric coefficient of ATP in the sucrose hydrolysis reaction (SUCRe). For a fixed capture efficiency, the growth cost in (A) was computed as the difference between the predicted growth under the histidine saturation conditions and that for a given histidine uptake rate. Similarly, the growth defect for a fixed capture efficiency in (B) was computed as the difference between the predicted growth rate when the ATP cost of sucrose hydrolysis is zero and that for a given ATP cost. As shown here, the model is not very much sensitive to histidine limitations. We found a similar pattern for other models of the yeast (Supplementary References 20 and 21; results not shown).

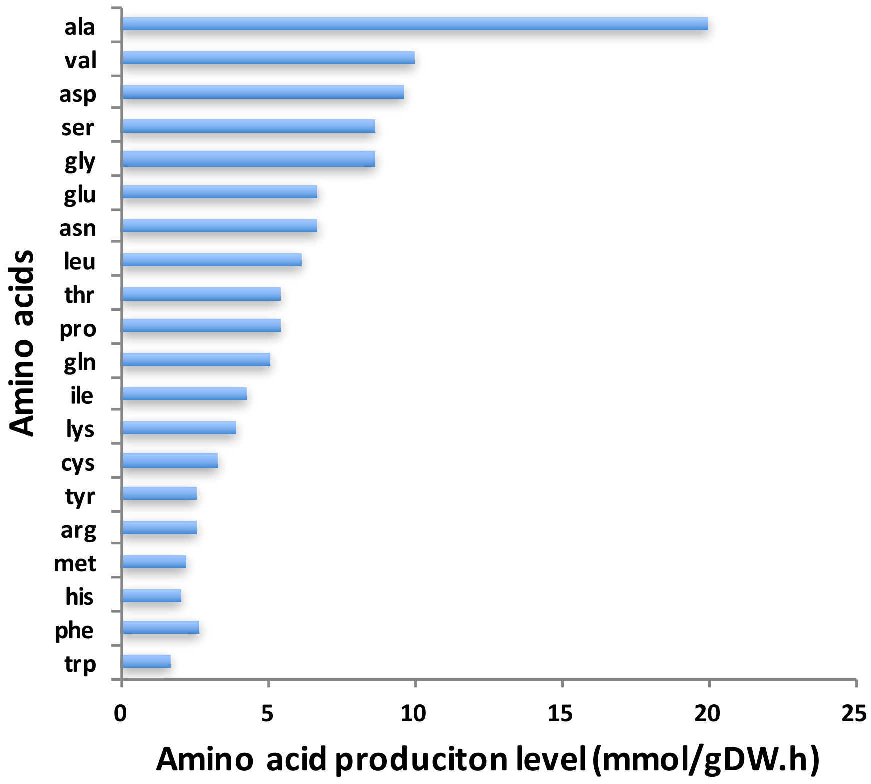

Supplementary Figure 15. Maximum production level of different amino acids under the anaerobic minimal conditions using the iJO1366 metabolic model of E. coli 25. Amino acids are shown here by their standard three-letter code.

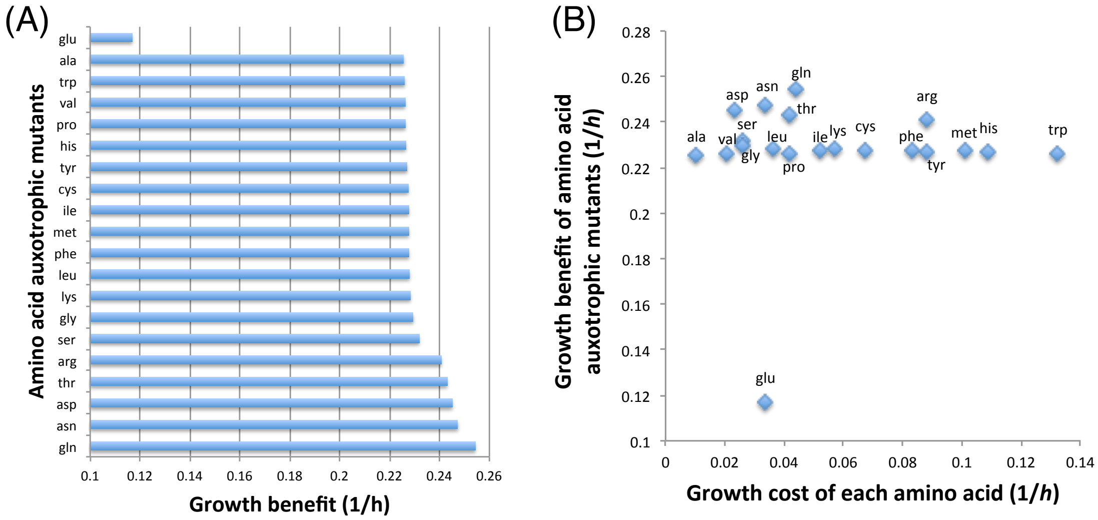

Supplementary Figure 16. Growth benefit vs. growth cost of amino acids. (A) *In silico* ranking of amino acid auxotrophic mutants according to the growth benefit per unit uptake of the respective amino acid. The growth benefit was quantified as the increase in growth rate (from a basal value of zero for the mutant) upon the uptake of 1 mmol/(gDW.h) of amino acid. (B) Growth benefit of amino acid auxotrophic mutant strains vs. the growth cost of the respective amino acid. Both growth gain and cost were computed for the uptake/production of 1 mmol/(gDW.h) of each amino acid.

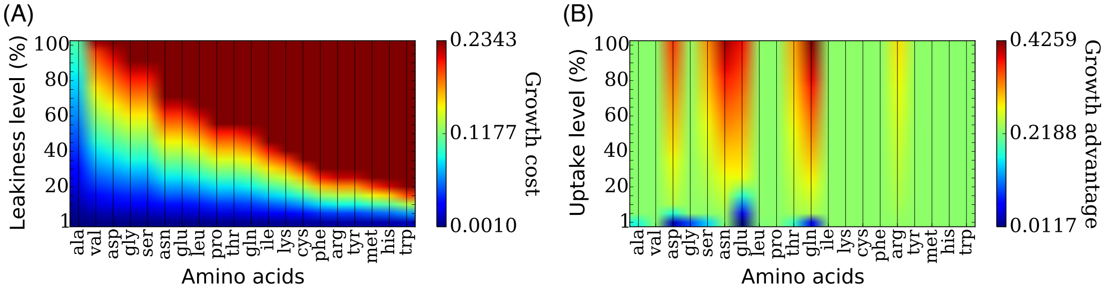

Supplementary Figure 17. Growth cost and growth benefit of amino acids as a function of leakiness and uptake levels. (A) The growth cost (1/*h*) of each amino acid for various leakiness (secretion) levels. Leakiness levels are described as the percentage of a maximum of 10 *mol/gDW.h* (see Supplementary Method 2 of details). Growth cost increases for all amino acids with increasing the leakiness level. (B) The growth benefit (1/*h*) of amino acid auxotrophic mutant strains as a function of the uptake level of the respective amino acids. Uptake levels are described as the percentage of a maximum of 10 *mol/gDW.h*. The growth rate of most mutant strains reaches a saturation point even for taking up 1% of this maximum implying that their growth rate will not increase any further by increasing the uptake levels (or leakiness level of a partner WT strain) beyond this threshold.

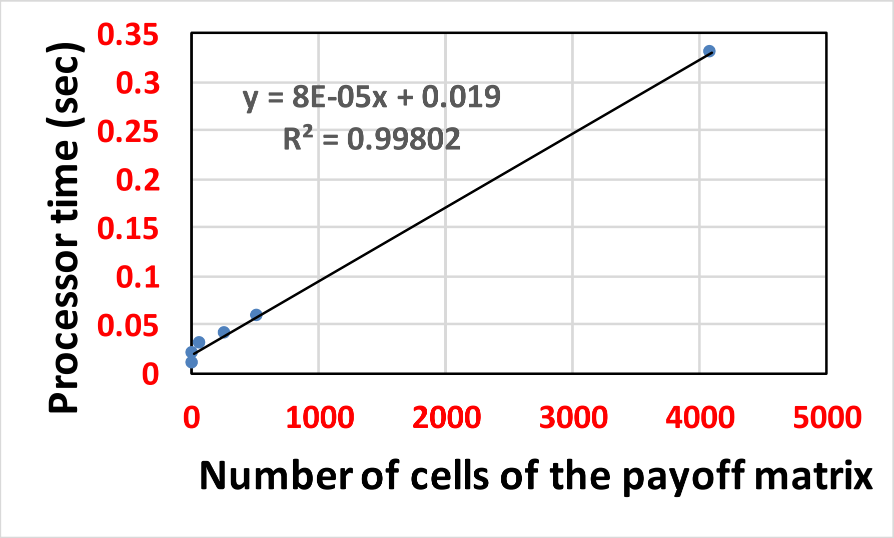

Case study	# of cells of the payoff matrix	Processor time (sec)
One leaky AA, Two-player game	4	0.02
Two leaky AAs, Two-player game	16	0.01
Two leaky AAs, Three-player game	64	0.03
Two leaky AAs, Four-player game	256	0.04
Three leaky AAs, Three-player game	512	0.06
Three leaky AAs, Four-player game	4096	0.33

Supplementary Figure 18. Processor times for gurobi optimization solver to solve NashEqu Finder for sample case studies of various complexity. Runtimes are reported for a compute node with dual fourteen-core 2.4GHz Intel Xeon E5-2680v4 CPUs and 256GB of RAM. Case studies are related to E. coli leaking different amino acids. As shown here, the processor time for solving NashEq Finder increases almost linearly with the number of cells of the payoff matrix.

Supplementary Table 1. A comparison of the key parameters of the Black Queen Hypothesis in the yeast sucrose hydrolysis and *E. coli* amino acid secretion systems.

**Property related to the Black Queen Hypothesis**	**Sucrose hydrolysis by *S. cerevisiae***	**Amino acnid secretion by *E. coli***
Leaked product	Glucose/fructose	Amino acids
Costly function	Invertase production	Amino acid synthesis
Level of publicizaiton/privatization	Capture efficiency (%)	Leakiness level (%)

Supplementary Note 1. Connections between this study and previous modeling and experimental work

1. Comparison between multi-species dynamic flux balance analysis approaches and the presented genome-driven evolutionary game theory framework

There are a number of differences between addressing community dynamics using the multi-species dynamic Flux Balance Analysis (dFBA) of metabolic models [1] (see also [2,3] for recent reviews of these methods) and that using the mechanistic evolutionary agme theory framework proposed in this study. Here, we aim to model only the 'evolutionary dynamics' of the system, that is, how the relative genotype abundances (frequencies, or community structure) change over time following the evolutionary game theory [4], and some microbial ecology literature [5]. It is worth noting that the simulation of *de novo* mutations and subsequent selection processes (which have been pursued in other studies [6]), is not the focus of our study. Evolutionary dynamics in evolutionary game theory is often modeled by using the Replicator Equation under the assumption of a constant population size [4] and captures the impact of genotype frequencies on the long-term state of the system (see Equations 6-8 in the main text). However, this equation is typically used as a phenomenological model with payoffs and reproductive fitness values obtained from intuition or from dedicated experiments. In this study, we proposed a new approach for the mechanistic modeling of evolutionary dynamics through integrating genome-scale metabolic networks and evolutionary game theory, where payoffs (and consequently reproductive fitness values) are obtained from genome-scale metabolic models.

Multi-species dFBA approaches, on the other hand, take into account the impact of variations in both genotype frequencies and genotype abundances and thus model the 'eco-evolutionary' dynamics of the system [5,7,8]. These approaches, however, require the knowledge of the uptake kinetics for the specific compounds that are taken up by each community member, which may not be always available.

2. Comparison with previous efforts to integrate metabolic networks and evolutionary game theory

While elementary mode analysis of metabolic networks [9] has been previously used in conjunction with evolutionary game theory to assess rate-yield tradeoffs in a single species [10], integrating constraint-based analysis of metabolic networks (i.e., COBRA-based methods [11]) with evolutionary game theory for the analysis of inter-species interactions has not been explored before. In this study we present (to our knowledge) the first example of such a framework.

3. Connections with previous experimental studies establishing synthetic cooperative exchanges

It is important to highlight a key difference between our *in silico* analyses and the previous experimental studies that report on establishing synthetic cross-feeding associations [12-16]. While these studies start with an initial population of complementary genotypes to assess whether they can grow in a co-culture, here we examined *in silico* the fundamental question of whether a one-way or two-way cooperative association can evolutionarily emerge from a population of prototrophic genotypes. In addition, our analyses show that cross-feeding can resist exploitation by other genotypes even in a homogenous environment, if such an association has already been established (see the main text and supplementary Figures. 10 and 11), which is consistent with previous experimental reports [17].

Supplementary Note 2. Metabolic inter-dependencies in invertase-producing *S. cerevisiae* system using a histidine auxotroph strain

Gore et al [18] used a histidine auxotroph yeast strain (lacking the gene *hisD*) as the producer genotype. This made it possible to increase the "cost of cooperation" experimentally by limiting the histidine concentration in the growth medium. However, since the incorporated ATP in the sucrose hydrolysis reaction (SUCRe) in the metabolic model serves as a proxy for the energetic cost of invertase production and secretion, we modeled variations in cooperation cost systematically and more explicitly in the analysis presented in the main text by changing the stoichiometric coefficient of ATP in the sucrose hydrolysis reaction (see Figure 2A of the main text and Section 3.1 in this document). Here, we present the results of the same analysis by using a histidine auxotroph producer genotype specifically reproducing *in silico* the system used in Gore et al [18]. Toward this end, an *in silico* producer strain was constructed by fixing the flux of reaction encoded by *hisD* in the *i*AZ900 model [19] at zero (see Supplementary Figure 13A). Furthermore, the stoichiometric coefficient of ATP in the sucrose hydrolysis reaction was tuned and fixed at a value such that the producer are 2.5% less fit than the non-producers as reported in [18] (see section 1 of section 2 of Supplementary Methods for details).

The identified Nash equilibria and equilibrium fraction of producers are given in Supplementary Figure 13B and 13C. Overall, the general patterns that are observed here are qualitatively in agreement with those in Figures 2B and 2C of the main text. The only difference is that the Prisoner's Dilemma game comprises only a small portion of the cost-capture efficiency landscape in Supplementary Figure 13B compared to that in Figure 2B of the main text. This is mostly due to the fact that the genome-scale metabolic models of the yeast [20,21] cannot fully recapitulate the growth defect (i.e., cooperation cost) due to histidine limitation in the absence of *hisD* gene (see Supplementary Figure 14).

Assessing the impact of the addition of glucose to the growth medium (Supplementary Figures 13D-F) also reveals patterns that are qualitatively in agreement with those in Figures 2E and 2F of the main text. The addition of glucose to the growth medium was modeled by allowing for the uptake of 5 mmol/(gDW.h) of glucose (for 10 mmol/(gDW.h) of sucrose uptake (using different values of glucose uptake leads to qualitatively similar results). When compared to Figure 2E, we observe that here a region of the Snowdrift game is still retained (Supplementary Figure 13E). One can observe that there is a noticeable decrease in the fraction of producers compared to the case where no additional glucose is provided in the Snowdrift game region (Supplementary Figures 13C and 13F). This is because in the presence of glucose in the extracellular environment, the non-producers are less dependent on producers for the availability of sugars, thereby increasing the average fitness of non-producers compared to the case where no glucose is provided. This makes producers more difficult to sustain i.e., they are either driven to extinction by non-producers (leading to a Prisoner's Dilemma game) or their equilibrium frequency decreases if a Snowdrift game region is still retained. These patterns are consistent with the reports by Gore et al [18] that increasing the glucose concentration in the growth medium leads to a decrease in the equilibrium fraction of producers and that this decrease can be so severe that eventually transforms the Mutually Beneficial or Snowdrift games to a Prisoner's Dilemma game.

Supplementary Note 3. Detailed analysis of the Nash equilibria and equilibrium genotype frequencies in *E. coli* strains secreting individual amino acids

Here, we analyze in more details the identified Nash equilibria and equilibrium genotype frequencies given in Figures 3B and 3D of the main text.

1. Analysis of the Nash equilibria

As noted in the main text and shown in Figure 3B of the main text, as the cost of amino acid production increases, the maximum leakiness level enabling (WT, MT) coexistence (i.e., Snowdrift game) decreases monotonically. Conversely, the size of the Prisoner's Dilemma region increases monotonically as the growth cost of amino acid production increases, with the smallest region being the one for alanine and the largest for tryptophan. This pattern is similar to the one observed for the yeast sucrose case (Figure 2B of the main text), where the role previously played by the ATP cost of sucrose hydrolysis is translated here into the amino acid-specific cost of leakiness. However, interestingly, a different, more complex pattern is observed in the *E. coli* case for the Mutually Beneficial region: in contrast to sucrose hydrolysis in yeast, here we have only a few cases where (WT, WT) appears as the Nash equilibrium with glutamate having the largest range of leakiness that supports such equilibria. For (WT, WT) to be a Nash equilibrium, a WT facing a WT must be fitter than a MT facing a WT. We reasoned that this would occur if the secreted amino acid was associated with a small growth cost to WT and/or a small growth advantage to the mutant. Indeed, for the most extreme case of glutamate, we found that the growth gain per unit uptake for a glutamate auxotroph mutant is significantly lower than all other amino acid auxotroph mutants (Supplementary Figure 16).

2. Analysis of the equilibrium genotype frequencies

As shown in Figure 3D of the main text, WT dominates in the Mutually beneficial game region, MT dominates in the Prisoner's Dilemma game region and WT and MT coexist in the Snowdrift game region. Here, we focus on analyzing the equilibrium

frequencies in the Snowdrift game region. As noted in the main text, the equilibrium fraction of MTs in the Snowdrift game region is the outcome of the complex balance between two factors (captured by the Nash equilibria of the game): (*i*) the growth cost of producing leaky amino acids and (*ii*) the growth benefit of auxotrophic mutant strains upon taking up those amino acids. The former increases as the leakiness level increases for all amino acids as expected (Supplementary Figure 17A). However, we do not observe a consistent pattern in the growth benefit of the mutant strains for all amino acids (Supplementary Figure 17B) consistent with previous reports [22]. In particular, we observed that the growth rate of MTs that appear only in a small equilibrium fraction in the Snowdrift game region has already reached a saturation point at very small uptake levels. This implies that any further increase in the leakiness levels in WT will not increase their growth rate. This causes the average fitness of these mutants to be lower than WT, which in turn leads to a higher frequency of WT in the Snowdrift game region.

Supplementary Methods

1. More details on NashEq Finder

Nash equilibrium is a central concept in game theory describing a state where no player has an incentive to change its current strategy because it cannot improve its payoff any further by doing so, if other players keep their strategies unchanged. As noted in the main text, we developed an optimization-based framework called *NashEq Finder* to identify the pure strategy Nash equilibria (NE) of a non-symmetric n-player game, given its payoff matrix (Sections 3 and 4 of this text provide detailed descriptions of how payoffs were computed for the case studies in this paper). In the following, we present an example of how NashEq Finder optimization formulation works using a simple two-player game, and also provide a preliminary assessment of the computational efficiency of the NashEq Finder algorithm.

1.1. An example showing how NashEq Finder optimization formulation works

Consider the following payoff matrix for a game with two players, ($p1$ and $p2$), where each player can either Cooperate (C) or Defect (D):

		$p2$: C	$p2$: D
$p1$	C	$a_{CC}^1 = 3, a_{CC}^2 = 3$	$a_{CD}^1 = 0, a_{CD}^2 = 6$
	D	$a_{DC}^1 = 5, a_{DC}^2 = 0.5$	$a_{DD}^1 = 1, a_{DD}^2 = 2$

We have:

$$P = \{C, D\}, \qquad Q = \{C, D\}$$

We also set $LB_1 = LB_2 = -1$.

It is easy to verify that $p1$ is better off defecting if $p2$ cooperates. The same is true if $p2$ defects as well. Similarly, $p2$ is always better off defecting no matter if $p1$ cooperates or defects. This implies that DD is the Nash equilibrium of the game (i.e.,

this is a Prisoner's Dilemma game). DD is the only cell of the payoff matrix that satisfies the conditions of the Nash equilibrium mathematically described with Constraints (6) and (7) in the NashEq Finder optimization formulation in the main text (note that w_{CD} is a binary variable and can assume only a value of zero or one):

Constraint (6):

$p = D, q = D$

For this constraint, the strategy of player $p2$ is fixed at D and we check whether taking D by player $p1$ is the best response to ($p2$,D):

$\max_{p' \in P}\{a^1_{p'D}\} = \max\{a^1_{CD}, a^1_{DD}\} = 1, \quad a^1_{DD} = 1$

$1 \geq 1w_{DD} + (-1)(1 - w_{DD}),$

which is satisfied for both $w_{DD} = 0$ and $w_{DD} = 1$.

Constraint (7):

$p = D, q = D$

For this constraint, the strategy of player $p1$ is fixed at D and we check whether taking D by player $p2$ is the best response to ($p1, D$):

$\max_{q' \in Q}\{a^2_{Dq'}\} = \max\{a^2_{DC}, a^2_{DD}\} = 2, \quad a^2_{DD} = 2$

$2 \geq 2w_{DD} + (-1)(1 - w_{DD}),$

which is satisfied for both $w_{DD} = 0$ $w_{DD} = 1$.

In this case we observe that both $w_{DD} = 0$ $w_{DD} = 1$ satisfy Constraints (6) and (7), however, since the objective function of the optimization problem maximizes sum of the binary variables, $w_{DD} = 1$ will be returned as the optimal value for w_{DD} upon solving the optimization problem.

Now, we verify that Constraints (6) and (7) are satisfied for other cells of the payoff matrix only if the value of the corresponding binary variable is zero. For example, assume the cell CD (i.e., $p1$ cooperates and $p2$ defects):

Constraint (6):

$p = C, q = D$

$\max_{p' \in P}\{a^1_{p'D}\} = 1, \quad a^1_{CD} = 0$

$0 \geq 1w_{CD} + (-1)(1 - w_{CD}),$

which is satisfied only for $w_{CD} = 0$

Constraint (7):

$p = C, q = D$

$\max_{q' \in Q}\{a^2_{Dq'}\} = 6, \quad a^2_{CD} = 6$

$6 \geq 6w_{CD} + (-1)(1 - w_{CD}),$

This inequality is satisfied for both $w_{CD} = 0$ and $w_{CD} = 1$. However, it is only $w_{CD} = 0$ that satisfies both Constraints (S1) and (S2). ☐

1.2. Computational efficiency of the NashEq Finder algorithm

Computing pure strategy Nash equlibria is known to be an NP-hard problem and it is PPAD-complete when considering mixed strategies as well [23,24]. As noted in the main text, we have provided, in Supplementary Software 1, a python script implementing NashEq Finder in its most general form to identify all pure strategy Nash equilibria of an n-player game. The runtime of NashEq primarily depends on the size of the payoff matrix (a function of number of players and the number of strategies each player can take) as one binary variable is assigned to each cell of the payoff matrix. Supplementary Figure 18 shows the required processor time to solve NashEq Finder for a number of sample simulations that we performed for *E. coli* leaking different number of amino acids. NashEq Funder complements previous mixed-integer programming approaches to identify the Nash equilibria of n-player games [25], however, a more comprehensive study is needed to find out how the computational efficiency of NashEq Finder is compared to that of the previous algorithms.

2. Computing the payoff values for the sucrose-hydrolysis populations of the *S. cerevisiae*

When growing on sucrose, *S. cerevisiae* produces the enzyme invertase that hydrolyzes sucrose and converts it glucose and fructose. This hydrolysis occurs in the periplasmic space where up to 99% of the glucose and fructose have been reported to diffuse away and serve as a public good, while the rest (quantified as the percent capture efficiency, e) can be retained by the cell [18]. Given that invertase production is metabolically and energetically costly, a mutant non-producer strategy may emerge through losing the invertase production genes. Gore et al [18] showed that depending on the cost of cooperation (i.e., the cost of invertase production) and on the glucose/fructose capture efficiency, a range of outcomes is possible: These include a Mutually Beneficial (Deadlock) game (where producers dominate), a Snowdrift game (where producers and non-producers coexist) or a Prisoner's Dilemma game (where non-producers dominate). As a proof-of-concept, we sought to examine whether a game theoretic model of this system based on genome-scale metabolism is able to reproduce the experimentally observed Nash equilibria. Here, sucrose hydrolysis (or invertase production) serves as the leaky function with percent leakiness $l = 100 - e$.

Now, we describe in detail how the payoffs of the game are computed for different ways of encountering producer (WT) and non-producer (MT) strains of the *S. cerevisiae* growing on sucrose. The python scripts used to generate the data and figures for this case study are fully available from the corresponding author upon request.

2.1. Definition of the *in silico* producer and non-producer strains

Non-producer: The non-producer strain lacks *suc2* gene, which is simulated by setting the lower and upper bounds on reaction SUCRe in the *i*AZ900 model [19] to zero.

Producer: The original sucrose hydrolysis reaction in the *i*AZ900 model (SUCRe) is as follows:

1.0 h2o_e + 1.0 sucr_e --> 1.0 glc_e + 1.0 fru_D_e

where, sucr_e, glc_e and fru_D_e stand for sucrose, glucose and fructose, respectively. This reaction is modified as follows to incorporate the ATP cost of sucrose hydrolysis as well as the glucose/fructose capture efficiency:

1.0 h2o_e + 1.0 sucr_e + s_{ATP} atp --> e glc_e + $(1-e)$ glc_secreted + e fru_e + $(1-e)$ fru_secreted + s_{ATP}adp + s_{ATP} pi

where, s_{ATP} and e denote the ATP cost of sucrose hydrolysis (i.e., the stoichiometric coefficient of ATP in SUCRe reaction) and glucose/fructose capture efficiency, respectively. In addition, glc_secreted and fru_secreted are two new compounds added to the metabolic model to model fractions of the glucose and fructose that are not captured by the cell. In order to allow the export of glc_secreted and fru_secreted out of the cell, we further added two exchange reactions for each of these compounds to the metabolic model:

EX_glc_secreted: glc_secreted <==>

EX_fru_secreted: fru_secreted <==>

Note that a positive and negative flux for an exchange reaction imply secretion and uptake, respectively. The *in silico* producer strain is defined as follows for two different ways of modeling the variations in cooperation cost:

(i) *Changes in invertase production cost is modeled by changing the ATP cost of sucrose hydrolysis*: In this case, we change the value of $s_{ATP,SUCRe}$ to model the changes in the cooperation cost.

(ii) *Changes in the invertase production cost is modeled by changing the histidine uptake rate*: This is to specifically reproduce Gore et al [18] system,

where they used a histidine auxotroph producer strain (Δ*hisD*), so as to experimentally increase the "cost of cooperation" by limiting the histidine concentration in the growth medium. Here, we first construct an *in silico* histidine auxotroph strain by setting the lower and upper bound for reaction IGPDH (imidazoleglycerol phosphate dehydratase) encoded by gene *hisD* to zero. We, next, manually adjusted $s_{ATP,SUCRe}$ such that the growth rate of a producer strain is 2.5% less than that of a non-producer strain as reported in [18]. However, since the *in silico* non-producer strain cannot grow on sucrose (due to having SUCRe reaction removed), we instead used the original *i*AZ900 model where SUCRe reaction does not contain an ATP cost as a proxy for the non-producer strain. We found with this analysis that $s_{ATP} = 0.115$, results in a growth rate for the producer strain, which is less than that for the non-producer by 2.5%.

All simulations were performed for aerobic minimal medium with a sucrose and oxygen uptake rates of $10 \frac{mmol}{gDW..h}$ and $2 \frac{mmol}{gDW.h}$, respectively, and with the flux of ATPM reaction fixed at $1 \frac{mmol}{gDW.h}$ [19,20].

2.2. Estimating additional metabolic parameters

We first need to determine a number of parameters before quantifying the payoff matrix of the game.

Death rate: A death rate was calculated requirements by using the following equation [1] for the case of an infeasible FBA problem implying that the available glucose in the growth medium cannot support maintenance ATP:

$$v_{death} = (q_s - m_s)Y_{\frac{x}{s}}$$

where, $q_s \left(\frac{mmol}{gDW.h}\right)$ is the uptake rate of the limiting substrate s (in this case glucose), $m_s \left(\frac{mmol}{gDW.h}\right)$ is the minimum uptake rate of limiting substrate s required to satisfy maintenance requirements and $Y_{\frac{x}{s}} \left(\frac{gDW}{mmol}\right)$ is the biomass yield of substrate s. m_s can

be easily found by solving an FBA problem where the objective function is to minimize the uptake rate of limiting substrate s. $Y_{\frac{x}{s}}$ can be also found by solving an FBA problem. Note that v_{death} takes a negative value since the cell undergoes death if $q_s < m_s$.

Growth cost of sucrose hydrolysis: We calculated a representative growth cost of sucrose hydrolysis for a fixed s_{ATP} by solving two FBA problems one for the original *i*AZ900 model (where no ATP cost is incorporated into the SUCRe reaction) and the other for the *in silico* producer strain (where ATP is incorporated into the SUCRe reaction but with $e = 1$). The growth cost of sucrose hydrolysis is then calculated as follows:

$$sucr\_hydr\_growth\_cost = -(v_{biomass}^{iAZ900} - v_{biomass}^{cooperator,e=1}).$$

Note that $sucr\_hydr\_growth\_cost$ always takes a negative value.

Glucose and fructose secretion rates: We first solve the following FBA problem for a producer strain alone, to find out the glucose and fructose secretion rates:

maximize $v_{biomass}$

subject to

$$\sum_{j \in J} s_{ij} v_j = 0, \qquad \forall i \in I, \qquad \text{(S1)}$$

$$LB_j \leq v_j \leq UB_j, \qquad \forall j \in J, \qquad \text{(S2)}$$

$$v_{EX\_sucr\_e} \geq -10, \qquad \text{(S3)}$$

$$v_{EX\_o2\_e} \geq -2, \qquad \text{(S4)}$$

$$v_{ATPM} = 1. \qquad \text{(S5)}$$

where, I and J denote the set of metabolites and reactions, s_{ij} is the stoichiometric coefficient of metabolite i in reaction j and v_j is the flux of reaction j. Furthermore, $v_{biomass}$, $v_{EX\_sucr\_e}$, $v_{EX\_o2\_e}$ and v_{ATPM} denote the flux of biomass reaction, sucrose exchange reaction, oxygen exchange reaction and ATP maintenance reaction,

respectively. Constraint (S1) represents the steady-state mass balance for each metabolite, Constraint (S2) imposes a lower (LB_j) and upper bound (UB_J) on each reaction flux, Unless specified otherwise, the lower bound is set to zero and -1000 for irreversible and reversible reactions, respectively, and the upper bound is set to 1000 for all reaction for all FBA problems throughout this paper. Constraints (S3) and (S4) set the bounds on the sucrose and oxygen uptake and finally Constraint (S5) fixes the flux of ATPM reaction at the specified value. The optimal values of reaction fluxes for $glc\_secreted$ and $fru\_secreted$ upon solving this problem are stored as $glc\_secreted\_flux$ and $fru\_secreted\_flux$. If this FBA problem is infeasible, $glc\_secreted\_flux$ and $fru\_secreted\_flux$ are set to $(1-e)10$, where 10 is the upper bounds on the sucrose uptake rate. Assigning a non-zero value to $glc\_secreted\_flux$ and $fru\_secreted\_flux$ in this case the goal was to guarantee that a partner (producer or non-producer) strain can still benefit from this cooperative behavior even when the cooperation is too costly.

With having these parameters determined, we can now compute the payoffs. Note that these payoffs are computed for the encounter of two single cells. The impact of cell type frequencies is accounted for when simulating the evolutionary dynamics using the Replicator's equation (see Methods in the main text). The following FBA formulations are presented for a fixed capture efficiency, e , and a fixed ATP cost of sucrose hydrolysis, s_{ATP} for case (i) in Section 3.1. These formulations can be readily adjusted for case (ii) as well.

2.3. Computing the payoffs for a producer vs. another producer

Here, we solve one FBA problem as follows:

maximize $v_{biomass}$

subject to

$$\sum_{j \in J} s_{ij} v_j = 0, \qquad \forall i \in I,$$

$$LB_j \leq v_j \leq UB_j, \qquad \forall j \in J,$$

$$v_{EX\_sucr\_e} \geq -10,$$

$$v_{EX\_o2\_e} \geq -2,$$

$$v_{EX\_glc\_e} \geq -glc\_secreted\_flux, \tag{S6}$$

$$v_{EX\_fru\_e} \geq -fru\_secreted\_flux, \tag{S7}$$

$$v_{ATPM} = 1.$$

where, EX_glc_e and EX_fru_e are the exchange reactions for glucose and fructose. Constraints (S6) and (S7) describe the fact that each single Producer strain can benefit from the secreted glucose and fructose by its partner Producer. The optimal value of $v_{biomass}$ is assigned as the payoff of a Producer vs. a Producer. If the problem is infeasible, the payoff is set to $v_{death} + sucr\_hydrol\_growth\_cost$ (note that both v_{death} and $sucr\_hydrol\_growth\_cost$ are both negative).

2.4. Computing the payoffs for a producer vs. a non-producer

The FBA problem for the producer is formulated as follows:

maximize $v_{biomass}$

subject to

$$\sum_{j \in J} s_{ij} v_j = 0, \qquad \forall i \in I,$$

$$LB_j \leq v_j \leq UB_j, \qquad \forall j \in J,$$

$$v_{EX\_sucr\_e} \geq -10,$$

$$v_{EX\_o2\_e} \geq -2,$$

$$v_{EX\_glc\_e} \geq 0 \tag{S8}$$

$$v_{EX\_fru\_e} \geq 0 \tag{S9}$$

$$v_{ATPM} = 1.$$

The difference between this FBA problem and that for producer vs. producer is that here we do not allow for the uptake of glucose and fructose as here the producer deals with a non-producer strain. As before, the optimal value of $v_{biomass}$ is assigned as the producer's payoff, if the FBA problem is solved to optimality, and is set to $v_{death} + sucr\_hydrol\_growth\_cost$, if it is infeasible.

The FBA for the non-producer is as follows:

maximize $v_{biomass}$

subject to

$$\sum_{j \in J} s_{ij} v_j = 0, \qquad \forall i \in I,$$

$$LB_j \leq v_j \leq UB_j, \qquad \forall j \in J,$$

$$v_{SUCRe} = 0 \tag{S10}$$

$$v_{EX\_sucr\_e} \geq -10,$$

$$v_{EX\_o2\_e} \geq -2,$$

$$v_{EX\_glc\_e} \geq -glc\_secreted\_flux,$$

$$v_{EX\_fru\_e} \geq -fru\_secreted\_flux,$$

$$v_{ATPM} = 1.$$

Here, the non-producer can benefit from the secreted glucose and fructose by its cooperative partner. The optimal value of $v_{biomass}$ is assigned as the non-producer's payoff, if the FBA problem is solved to optimality. Otherwise, the payoff of the non-producers is set to v_{death} (note that the non-producer does not incur the cost of sucrose hydrolysis).

2.5. Computing the payoffs for a non-producer vs. another non-producer

There is no need to solve an FBA problem in this case, because it is known *a priori* that an FBA problem for a non-producer vs. a non-producer will be infeasible because the ATP maintenance requirements (described by $v_{ATPM} = 1$ [20]) cannot be satisfied due to the absence of glucose/fructose. The payoff of the non-producer is set to v_{death} in this case.

3. Computing the payoff values for amino acid-secreting *E. coli* strains

Here, we describe the details of how the payoffs are computed for games involving *E. coli* strains that are auxotroph and/or leaky for certain amino acids (the python scripts used to generate the data and figures for this case study are fully available from the corresponding author upon request). It is worth noting that despite the inherent differences between sucrose hydrolysis by yeast and amino acid secretion by *E. coli* both systems can be studied in the light of the Black Queen Hypothesis. A side-by-side comparison of the key properties related to the Black Queen Hypothesis in these two systems is given in Supplementary Table 1.

All simulations were performed using the *i*JO1366 metabolic model of *E. coli* [26] under the anaerobic minimal conditions with glucose as the sole carbon source following previous experimental studies [12,27]. [12]The lower bound on the exchange flux of glucose was set to $-10\,\frac{mmol}{gDW}$ and that for the rest of the compounds in the medium was set to $-1000\,\frac{mmol}{gDW.h}$ [28]. The lower bound for the rest of exchange fluxes as well as that for irreversible reactions was set to zero. The upper bound for all reactions was set to $1000\,\frac{mmol}{gDW.h}$, unless otherwise specified. The flux of ATP maintenance reactions was also set to $8.39\,\frac{mmol}{gdw.h}$.

3.1. Identifying the maximum production levels of amino acids

We solved an FBA problem maximizing the exchange flux of each amino acid, one at a time, in order to identify the maximum possible amino acid production levels by a wild-type strain under the uptake and aeration conditions mentioned above. The results of this analysis are summarized in Supplementary Figure 15. One can observe that except for ala (alanine), the maximal production level of the rest of amino acids is less than or equal to $10\,\frac{mmol}{gDW.h}$. Therefore, we decided to use a universal maximal production level of $10\,\frac{mmol}{gDW.h}$ for all amino acids. For the purpose of standardizing the presented results across different amino acids, the leakiness levels were expressed as a certain percentage of this maximum.

3.2. Construction of the *in silico* strains

Auxotrophy for one or more amino acids is imposed by setting the lower and upper bounds for reactions corresponding to the genes responsible for the synthesis of those amino acid to zero. A list of genes coding for the synthesis of all amino acids and their corresponding reactions in the model are given in Supplementary Data 2. We considered only gene mutations that lead to the auxotrophy only for one amino acid in 01 and 10 genotypes and to auxotrophy for both amino acids in 00 a genotype according to the metabolic model. Note that while the composition of biomass in metabolic network models is constant (and captured by biomass reaction) across all mutants arising from the same wild-type strain, it is quite possible for the mutant strains to have a biomass composition slightly different from that of the wild-type. Future studies may take into account this consideration to provide a more realistic representation of these mutant strains. Amino acid leakiness was imposed in the model by setting the lower bounds of the corresponding exchange reactions to a certain percentage of the maximum value of $10\,\frac{mmol}{gDW.h}$. it should be noted that the impact of leaking an amino acid at a particular leakiness level on the fitness (growth) of that genotype depends not only on the cost of synthesizing that amino acid but also on its transport cost, in case the transport of this amino acid is metabolically or energetically costly (all these costs are captured by the metabolic network model).

3.3. Determining the net export and uptake level of each amino acid by each community member

Here, we assume that the leakiness level of each amino acid is the same across all genotypes in the community that are leaky for that amino acid given that all mutant genotypes arise from a wild-type genotype. For example, if the leakiness level of amino acid AA is 25%, all genotypes that are not auxotroph for this AA will secrete $0.25{\times}10\,\frac{mmol}{gDW.h}$ of AA. Now, let p be the leakiness level (in percent) of a given amino acid AA (corresponding to a production level of $\left(\frac{p}{100}\right){\times}10\,\frac{mmol}{gDW.h}$), N be the total number of genotypes in a given encounter (e.g., $N = 2$ for pairwise interacitons and

$N = 3$ for the encounter of three genotypes), and N_{AA} denote the total number of genotypes in the game secreting amino acid AA. Then, the net uptake rate of an amino acid AA for a genotype auxotroph for AA is determined as follows:

$$v_{net,uptake} = \frac{N_{AA}\left(\frac{10p}{100}\right)}{N-1} \tag{S11}$$

Similarly, the net secretion (export) level of amino acid AA by a genotype in the game that is leaky for this amino acid is determined as follows:

$$v_{net,export} = \frac{10p}{100} - \frac{(N_{AA}-1)\left(\frac{10p}{100}\right)}{N-1} \tag{S12}$$

Note that if the net export turns out to be negative, it implies uptake. This may happen for three or more leaky traits.

The main assumption underlying these relations is that a genotype that is leaky for an amino acid does not have access to part of the amino acid that is secreted out but it can access those secreted by other genotypes in the community. This assumption is to impose the notion that the private benefit is the portion of the amino acid that is retained by producing cells and the rest will serve as the public good. In addition, we assume, for simplicity, that any secreted amino acid by a given genotype is equally shared among all other the genotypes involved in the game (community). This is a reasonable assumption in a homogeneous environment, which is the focus of this study. Note that these assumptions do not affect the generality of our framework and can be easily relaxed, if needed. Some examples of how these relations work are given in the following, where 1 and 0 represent cases in which a genotype is leaky or auxotroph for a given amino acid, respectively.

Examples:

(i) Pairwise interactions and one leaky trait (amino acid AA):

1 vs. 1: $N = 2$, $N_{AA} = 2$, $v^{1}_{net,export} = \frac{10p}{100} - \frac{(2-1)\left(\frac{10p}{100}\right)}{2-1} = 0$

This implies that the same amount that is exported by genotype 1 is taken up from its partner genotype 1.

(ii) Pairwise interactions and two leaky traits (with amino acid AA being the second trait):

11 vs. 11: $N = 2,\ \ N_{AA} = 2,\ \ v^{11}_{net,export} = \frac{10p}{100} - \frac{(2-1)\left(\frac{10p}{100}\right)}{2-1} = 0$

11 vs. 10 (with amino acid AA being the second trait):

$N = 2,\ N_{AA} = 1,\ v^{11}_{net,export} = \frac{10p}{100} - \frac{(1-1)\left(\frac{10p}{100}\right)}{2-1} = \frac{10p}{100},\ \ v^{10}_{net,uptake} = \frac{1\left(\frac{10p}{100}\right)}{2-1} = \frac{10p}{100}$

11 vs. 11 vs. 10 (with amino acid AA being the second trait):

$N = 3,\ N_{AA} = 2,\ v^{11}_{net,export} = \frac{10p}{100} - \frac{(2-1)\left(\frac{10p}{100}\right)}{3-1} = 0.5\,\frac{10p}{100},\ \ v^{10}_{net,uptake} = \frac{2\left(\frac{10p}{100}\right)}{3-1} = \frac{10p}{100}$

The implies that the net export of AA by 11 genotypes is only $0.5\,\frac{10p}{100}$ because they secrete $1\,\frac{10p}{100}$ but can take up $0.5\,\frac{10p}{100}$ from another 11. Furthermore the net uptake of AA by 10 genotype is $\frac{10p}{100}$ composed of two $0.5\,\frac{10p}{100}$, each secreted by one of the two 11 genotypes.

3.4. Formulation of the FBA problems to quantify the payoffs

One FBA problem is solved for each distinct genotype in a pairwise or higher-order interaction. For example, if computing the payoffs for the encounter of three genotypes 11, 11 and 10, one needs to solve only one FBA problem for 11 genotypes and one for the 10 genotype. The list of exchanged amino acids and their leakiness levels are provided as inputs. The general form of the FBA problem that is solved for a given genotype k in the game (community) is as follows:

maximize $v^k_{biomass}$

subject to

$$\sum_{j \in J^k} s^k_{ij} v^k_j = 0, \qquad \forall i \in I^k,$$

$$LB_j^k \leq v_j^k \leq UB_j,^k \qquad \forall j \in J^k,$$

$$v_{EX\_glc\_e} \geq -10, \tag{S13}$$

$$v_{j^*}^k = 0, \qquad \forall j^* \in KO^k \tag{S14}$$

$$v_{EX\_i\_e}^k \geq v_{net,export,i}^k, \qquad \forall i \in I^{leaky,k} \tag{S15}$$

$$v_{EX\_i\_e}^k \geq -v_{net,uptake,i}^k, \qquad \forall i \in \{i | i \notin I^{leaky,k} \;\&\; i \in I^{leaky,k'}, \forall k' \neq k\}, \tag{S16}$$

$$v_{ATPM} = 8.39. \tag{S17}$$

where, all parameters and variables defined for an FBA problem in Section 3 are extended here by addition of a superscript k denoting that they belong to the community member k. Here, KO^k and $I^{leaky,k}$ denote the set of reactions corresponding to the knocked out genes and the set of leaky metabolites for genotype k, respectively. Constraint (S14) sets to zero the flux of reactions corresponding to the specific gene mutations in the genotype under consideration. Constraints (S15) and (S16) impose the bounds on the export rate of metabolites for which genotype k is leaky and on the uptake rate of metabolites for which it is auxotroph, where $v_{net,export,i}^k$ and $v_{net,uptake,i}^k$ are obtained from Equations (S11) and (S12), respectively. Constraint (S17) sets the flux of maintenance ATP reaction in the model to 8.39 $\frac{mmol}{gDW.h}$ following previous studies [28]. The general FBA formulation can be customized for any desired genotypes by adjusting KO^k and $I^{leaky,k}$. If the FBA problem is solved to optimality, the obtained biomass flux is assigned as the payoff of the corresponding genotype. Otherwise, the payoff is set to $death\_rate$ (a negative value), which is computed beforehand the same way it was described in section 2 of Supplementary Methods.

Supplementary References

1 Zhuang, K. *et al.* Genome-scale dynamic modeling of the competition between Rhodoferax and Geobacter in anoxic subsurface environments. *ISME J* **5**, 305-316, doi:10.1038/ismej.2010.117 (2011).

2 Zomorrodi, A. R. & Segrè, D. Synthetic Ecology of Microbes: Mathematical Models and Applications. *J Mol Biol*, doi:10.1016/j.jmb.2015.10.019 (2015).

3 Biggs, M. B., Medlock, G. L., Kolling, G. L. & Papin, J. A. Metabolic network modeling of microbial communities. *Wiley Interdisciplinary Reviews-Systems Biology and Medicine* **7**, 317-334, doi:10.1002/wsbm.1308 (2015).

4 Nowak, M. A. *Evolutionary dynamics : exploring the equations of life*. (Belknap Press of Harvard University Press, 2006).

5 Sanchez, A. & Gore, J. feedback between population and evolutionary dynamics determines the fate of social microbial populations. *PLoS Biol* **11**, e1001547, doi:10.1371/journal.pbio.1001547 (2013).

6 Elena, S. F. & Lenski, R. E. Evolution experiments with microorganisms: the dynamics and genetic bases of adaptation. *Nat Rev Genet* **4**, 457-469, doi:10.1038/nrg1088 (2003).

7 Post, D. M. & Palkovacs, E. P. Eco-evolutionary feedbacks in community and ecosystem ecology: interactions between the ecological theatre and the evolutionary play. *Philos Trans R Soc Lond B Biol Sci* **364**, 1629-1640, doi:10.1098/rstb.2009.0012 (2009).

8 Rauch, J., Kondev, J. & Sanchez, A. Cooperators trade off ecological resilience and evolutionary stability in public goods games. *J R Soc Interface* **14**, doi:10.1098/rsif.2016.0967 (2017).

9 Trinh, C. T., Wlaschin, A. & Srienc, F. Elementary mode analysis: a useful metabolic pathway analysis tool for characterizing cellular metabolism. *Appl Microbiol Biotechnol* **81**, 813-826, doi:10.1007/s00253-008-1770-1 (2009).

10 Schuster, S., de Figueiredo, L. F., Schroeter, A. & Kaleta, C. Combining metabolic pathway analysis with Evolutionary Game Theory: explaining the occurrence of low-yield pathways by an analytic optimization approach. *Biosystems* **105**, 147-153, doi:10.1016/j.biosystems.2011.05.007 (2011).

11 Becker, S. A. *et al.* Quantitative prediction of cellular metabolism with constraint-based models: the COBRA Toolbox. *Nat Protoc* **2**, 727-738, doi:10.1038/nprot.2007.99 (2007).

12 Wintermute, E. H. & Silver, P. A. Emergent cooperation in microbial metabolism. *Mol Syst Biol* **6**, 407, doi:10.1038/msb.2010.66 (2010).

13 Mee, M. T., Collins, J. J., Church, G. M. & Wang, H. H. Syntrophic exchange in synthetic microbial communities. *Proc Natl Acad Sci U S A* **111**, E2149-2156, doi:10.1073/pnas.1405641111 (2014).

14 Shou, W., Ram, S. & Vilar, J. M. Synthetic cooperation in engineered yeast populations. *Proc Natl Acad Sci U S A* **104**, 1877-1882, doi:10.1073/pnas.0610575104 (2007).

15 Harcombe, W. Novel cooperation experimentally evolved between species. *Evolution* **64**, 2166-2172, doi:10.1111/j.1558-5646.2010.00959.x (2010).

16 Hoek, T. A. *et al.* Resource Availability Modulates the Cooperative and Competitive Nature of a Microbial Cross-Feeding Mutualism. *PLoS Biol* **14**, e1002540, doi:10.1371/journal.pbio.1002540 (2016).
17 Pande, S. *et al.* Fitness and stability of obligate cross-feeding interactions that emerge upon gene loss in bacteria. *ISME J* **8**, 953-962, doi:10.1038/ismej.2013.211 (2014).
18 Gore, J., Youk, H. & van Oudenaarden, A. Snowdrift game dynamics and facultative cheating in yeast. *Nature* **459**, 253-256, doi:10.1038/nature07921 (2009).
19 Zomorrodi, A. R. & Maranas, C. D. Improving the iMM904 S. cerevisiae metabolic model using essentiality and synthetic lethality data. *BMC Syst Biol* **4**, 178, doi:10.1186/1752-0509-4-178 (2010).
20 Mo, M. L., Palsson, B. O. & Herrgård, M. J. Connecting extracellular metabolomic measurements to intracellular flux states in yeast. *BMC Syst Biol* **3**, 37, doi:10.1186/1752-0509-3-37 (2009).
21 Chowdhury, R., Chowdhury, A. & Maranas, C. D. Using Gene Essentiality and Synthetic Lethality Information to Correct Yeast and CHO Cell Genome-Scale Models. *Metabolites* **5**, 536-570, doi:10.3390/metabo5040536 (2015).
22 D'Souza, G. *et al.* Less is more: selective advantages can explain the prevalent loss of biosynthetic genes in bacteria. *Evolution* **68**, 2559-2570, doi:10.1111/evo.12468 (2014).
23 Gilboa, I. & Zemel, E. Nash and correlated equilibria: some complexity considerations. *Games and Economic Behavior* **1**, 80-93 (1989).
24 Daskalakis, C., Goldberg, P. W. & Papadimitriou, C. H. The Complexity of Computing a Nash Equilibrium. *Communications of the Acm* **52**, 89-97, doi:10.1145/1461928.1461951 (2009).
25 Wu, Z. T., Dang, C. Y., Karimi, H. R., Zhu, C. A. & Gao, Q. A Mixed 0-1 Linear Programming Approach to the Computation of All Pure-Strategy Nash Equilibria of a Finite n-Person Game in Normal Form. *Mathematical Problems in Engineering*, doi:10.1155/2014/640960 (2014).
26 Orth, J. D. *et al.* A comprehensive genome-scale reconstruction of Escherichia coli metabolism--2011. *Mol Syst Biol* **7**, 535, doi:10.1038/msb.2011.65 (2011).
27 Zomorrodi, A. R., Islam, M. M. & Maranas, C. D. d-OptCom: Dynamic multi-level and multi-objective metabolic modeling of microbial communities. *ACS Synth Biol* **3**, 247-257, doi:10.1021/sb4001307 (2014).
28 Feist, A. M. *et al.* A genome-scale metabolic reconstruction for Escherichia coli K-12 MG1655 that accounts for 1260 ORFs and thermodynamic information. *Mol Syst Biol* **3**, 121, doi:10.1038/msb4100155 (2007).
