# Supplementary material for: Genome-driven evolutionary game theory helps understand the rise of metabolic interdependencies in microbial communities
Source: Nat Commun. 2017 Nov 16;8:1563. doi: 10.1038/s41467-017-01407-5 (PMC5691134; doi:10.1038/s41467-017-01407-5)
Supplement: Supplementary file 2 — Description of Additional Supplementary Files [file 41467_2017_1407_MOESM2_ESM.pdf]

Description of Additional Supplementary Files

File Name: Supplementary Data 1
Description: List of genes removed in the model to create auxotrophy for each amino acid.

File Name: Supplementary Data 2
Description: Original data values used in Figure 3C.

File Name: Supplementary Data 3
Description: List of amino acid pairs for which the region of sustainable leakiness levels (or a subset of this region) does not include a unidirectional dependency.

File Name: Supplementary Software 1
Description: Python script implementing the NashEq Finder algorithm
